# SDHA Deficiency in Hepatocellular Carcinoma Promotes Tumor Progression through Succinate-Induced M2 Macrophage Polarization

**DOI:** 10.32604/or.2025.073179

**Published:** 2026-01-19

**Authors:** Xinyang Li, Luyuan Ma, Chuan Shen, Ruolan Gu, Shilong Dong, Mingjie Liu, Ying Xiao, Wenpeng Liu, Yuexia Liu, Caiyan Zhao

**Affiliations:** 1 Department of Infectious Diseases, Hebei Medical University Third Hospital, Shijiazhuang, 050000, China; 2 Clinical Research Center for Infectious Disease of Hebei Province, Shijiazhuang, 050000, China; 3 Hebei Key Laboratory for Diagnosis, Treatment, Emergency Prevention and Control of Critical Infectious Diseases, Shijiazhuang, 050000, China

**Keywords:** Hepatocellular carcinoma, metabolic reprogramming, tumor microenvironment, succinate, M2 macrophage, succinate dehydrogenase complex subunit A (SDHA), G protein-coupled receptor 91 (GPR91)

## Abstract

**Background:**

Hepatocellular carcinoma (HCC) is an aggressive and lethal malignancy. Metabolic reprogramming dynamically remodels the tumor microenvironment (TME) and drives HCC progression. This study investigated the mechanism through which metabolic reprogramming remodels the TME in HCC.

**Methods:**

HCC patient transcriptome data were subjected to bioinformatics analysis to identify differentially expressed genes and immune infiltration status. Immunohistochemical analysis was performed to determine the correlation between succinate dehydrogenase complex subunit A (SDHA) expression and M2 macrophage infiltration. SDHA-knockdown or SDHA-overexpressing HCC cells were used for *in vitro* experiments, including co-culturing, flow cytometry, and enzyme-linked immunosorbent assay. Western blotting assay, functional assays, and subcutaneous tumor model mice were used to elucidate the molecular mechanisms underlying succinate-mediated HCC cell-macrophage interactions in the TME.

**Results:**

Higher infiltration of M2 macrophages correlated with worse prognosis in HCC patients. SDHA was downregulated in HCC tumor tissues and showed a negative correlation with M2 macrophage infiltration. SDHA knockdown promoted M2 macrophage polarization, whereas SDHA overexpression reversed this effect. Mechanistically, SDHA deficiency in HCC cells induced succinate accumulation, which promoted M2 macrophage polarization by activating the G protein-coupled receptor 91 (GPR91)/signal transducer and activator of transcription 3 (STAT3) pathway. Concurrently, succinate stimulation enhanced mitochondrial oxidative phosphorylation in M2 macrophages, thereby promoting HCC progression. Serum succinate levels were elevated in HCC patients. The receiver operating characteristic curve analysis indicated that serum succinate is a promising diagnostic marker for HCC (area under the curve = 0.815).

**Conclusion:**

SDHA deficiency leads to succinate accumulation, which promotes M2 macrophage polarization through the GPR91/STAT3 pathway, thereby facilitating HCC progression. Based on these findings, serum succinate could be a promising diagnostic biomarker for HCC.

## 1 Introduction

Hepatocellular carcinoma (HCC) is one of the most prevalent malignant tumors worldwide [1]. Surgical resection and liver transplantation are the most effective curative treatment options for early-stage HCC patients [2]. However, the majority of HCC patients are diagnosed at an advanced stage and experience a 5-year survival rate of <18% despite systemic therapies [3]. Therefore, it is essential to elucidate the molecular mechanisms underlying HCC to discover novel therapeutic targets and prognostic biomarkers.

The emergence of immunotherapy has highlighted the pivotal role of the tumor microenvironment (TME) in cancer progression and treatment response [4]. The TME comprises cellular and non-cellular components surrounding tumor cells and plays an integral role in tumor initiation, development, metastasis, and therapeutic efficacy [5]. Tumor-associated macrophages (TAMs) constitute a key cellular component within the complex ecosystem of the TME [6]. They exhibit functional and phenotypic plasticity, polarizing into either proinflammatory M1 or immunosuppressive M2 phenotypes in response to microenvironmental signals, thereby exerting distinct biological effects throughout tumor progression [7]. TAMs predominantly display an M1 phenotype during early stages of tumorigenesis. M1-polarized TAMs promote antitumor immunity by enhancing T-cell infiltration and activation [8]. TAMs exhibit an M2 phenotype during tumor progression and metastasis [9]. M2-polarized macrophages facilitate immune evasion and tumor progression by secreting anti-inflammatory cytokines and chemokines, which suppress T-cell activity [10]. A higher proportion of M1 macrophages correlates with improved immunotherapy outcomes. Therefore, strategies targeting the reduction of M2 macrophage proportion or infiltration in the TME enhance antitumor immunity [11].

Tumor metabolic reprogramming, a hallmark of cancer, involves significant metabolic alterations in cancer cells to support their rapid proliferation. This process simultaneously reshapes the TME and modulates the function and polarization of TAMs [12]. Mitochondria play a central role in regulating cellular metabolism. Disrupted mitochondrial function drives tumor metabolic reprogramming and modulates the functions of immune cells by remodeling the TME, thus functioning as a crucial hub in the tumor metabolism-immune crosstalk network [13]. Specifically, tumor-derived metabolites, including lactate, fatty acids, succinate, α-ketoglutarate and glutamine, promote M2 polarization of TAMs through lactylation, peroxisome proliferator-activated receptor γ signaling, and metabolic reprogramming, whereas high potassium levels enhance the immunosuppressive function of TAMs within the TME [14,15]. Overall, these metabolic shifts promote the polarization of the TAMs toward a pro-tumorigenic M2 phenotype and create a favorable microenvironment for tumor progression [16]. Therefore, understanding the interplay between tumor metabolic reprogramming and TAMs may reveal novel therapeutic targets for cancer treatment and potential cancer immunotherapeutic strategies for future application.

In the present study, we investigated the mechanisms by which metabolic reprogramming in the TME of HCC tissues remodels the immune microenvironment. Specifically, we analyzed the infiltration of M2 macrophages in HCC tissues and identified succinate dehydrogenase (SDH) complex subunit A (SDHA) as a key metabolism-related differentially expressed gene significantly associated with M2 macrophages. SDHA, a pivotal component of mitochondrial oxidative phosphorylation (OXPHOS) in the tricarboxylic acid cycle (TCA), enhances HCC cell proliferation [17]. However, its potential immunomodulatory functions within the TME of HCC tissues remain unexplored. Therefore, this study also investigated whether SDHA deficiency in HCC influences M2 macrophage polarization, metabolic reprogramming in the TME, and tumor progression in HCC. Overall, our aim was to understand the metabolic regulation of immunity in HCC to identify promising novel targets for developing metabolism-targeted tumor immunotherapeutic strategies.

## 2 Materials and Methods

### 2.1 HCC Datasets

Genetic data and clinical characteristics of HCC samples were obtained from the genomic data commons the cancer genome atlas (TCGA) liver cancer project on the Xena (https://xenabrowser.net/datapages/, accessed on 01 January 2025). This included 345 HCC patients with complete clinical data and a survival period exceeding 30 days. The gene microarray dataset GSE76427, comprising 115 HCC samples and 52 adjacent non-tumor samples, was downloaded from the Gene Expression Omnibus (GEO) database (https://www.ncbi.nlm.nih.gov/geo/, accessed on 01 January 2025) [18]. The gene expression profiles were transformed and normalized by R 4.4.2 for further data analysis. Differentially expressed genes (DEGs) between the cancerous and adjacent normal tissues were evaluated using the tumor immune estimation resource version 2.0 (TIMER 2.0) database (https://compbio.cn/timer2/, accessed on 01 January 2025).

### 2.2 Immune Cell Infiltration Analysis and Key Gene Identification

Cell-type identification by estimating relative subsets of RNA transcripts (CIBERSORT) was used to evaluate the tumor-infiltration of immune cell types in HCC samples from the TCGA and GSE76427 datasets. Subsequently, the prognostic significance of the tumor-infiltrating immune cells was analyzed using the ‘survival’ package 3.7. DEGs between high and low M2 macrophage infiltration groups were identified using the ‘limma’ package 3.62.2 based on the criteria of an absolute fold change ≥1.2 and a *p* value < 0.05. In this study, a comprehensive literature screening was conducted to identify metabolism-related genes (MRGs). A total of 2752 MRGs were ultimately obtained, which reportedly encode all known human metabolic enzymes and small molecule transporters. Subsequently, we focused on the metabolism-related DEGs and performed functional annotation analysis using the Database for Annotation, Visualization and Integrated Discovery (DAVID) (https://davidbioinformatics.nih.gov/, accessed on 01 January 2025) to elucidate their biological roles in biological process (BP), cellular component (CC), and molecular function (MF). A protein-protein interaction (PPI) network was then constructed using the search tool for the retrieval of interacting genes/proteins (STRING) database (https://cn.string-db.org/, accessed on 01 January 2025) with these metabolism-related DEGs. The PPI network was analyzed using Cytoscape 3.10.2 to identify the three most highly connected genes as key DEGs. Then, their prognostic relevance was analyzed using the Kaplan–Meier plotter database (https://kmplot.com/analysis/index.php?p=home, accessed on 01 January 2025). Gene set enrichment analysis (GSEA) was performed using the ‘clusterProfiler’ 4.14.6 to determine the signaling pathways associated with G protein-coupled receptor 91 (GPR91).

### 2.3 Clinical HCC Samples

The following clinical samples were obtained from the Third Hospital of Hebei Medical University: (1) 52 paired HCC tissues and adjacent normal tissues, including 17 freshly collected tissue samples, which were immediately snap-frozen in liquid nitrogen and stored at −80°C for subsequent analysis; (2) serum samples from 54 HCC patients and 18 healthy volunteers. Written informed consent was obtained from all participants. The study protocol was approved by the Institutional Ethics Committee of the Hebei Medical University Third Hospital (No. 2024-012-1).

### 2.4 Cell Culture

The following cell lines were used: the human normal liver cell line THLE-2 (#CL-0833, Pricella, Wuhan, China); the HCC cell lines MHCC97H (#STCC10113P, Servicebio, Wuhan, China), Hep3B (#STCC10103P, Servicebio, Wuhan, China), HepG2 (#STCC10114P, Servicebio, Wuhan, China), HuH7 (#STCC10102P, Servicebio, Wuhan, China), and HCC-LM3 (#STCC10111P, Servicebio, Wuhan, China); the mouse HCC cell line Hepa1-6 (#STCC20016P, Servicebio, Wuhan, China); and the human monocytic cell line THP-1 (#CL-0233, Pricella, Wuhan, China). THLE-2 and THP-1cells were cultured in special medium (THLE-2: #CM-0833, THP-1: #CM-0233, Procell, Wuhan, China). The HCC cell lines were cultured in DMEM (#C11995500BT, Gibco, Invitrogen, Thermo Fisher Scientific, Carlsbad, CA, USA) supplemented with 10% fetal bovine serum (#AUS-01S, Cellbox, Suzhou, China) and 1% penicillin-streptomycin (#PB180120, Pricella, Wuhan, China). The culture environment was set at 37°C in an incubator containing 5% CO_2_. All cell cultures underwent validation via short tandem repeat profiling and confirmed absence of mycoplasma contamination.

### 2.5 Cell Transfections

Gene knockdown was performed by transfecting Hep3B cells with SDHA-siRNA (siSDHA-1 and siSDHA-2) or a negative control siRNA (siNC), both designed and synthesized by JTSBIO Co., Ltd. (Wuhan, China), using Lipofectamine™ RNAiMAX (#13778030, Invitrogen, Thermo Fisher Scientific, Carlsbad, CA, USA). For gene overexpression, MHCC97H cells were transfected with an SDHA overexpression plasmid or an empty vector control (EV), which were designed and synthesized by Biomed Gene Technology Co., Ltd. (Beijing, China), using Lipofectamine® 2000 (#11668030, Invitrogen, Thermo Fisher Scientific, Carlsbad, CA, USA). The transfection efficacies were subsequently verified by quantitative reverse transcription PCR (qRT-PCR) and western blot (WB) analyses.

### 2.6 qRT-PCR

Total RNA was extracted from human tissues, a panel of cell lines (THLE-2, MHCC97H, Hep3B, HepG2, HuH7, and HCC-LM3), and macrophages using TRIzol reagent (#15596018, Invitrogen, Thermo Fisher Scientific, Carlsbad, CA, USA). RNA concentration and purity were determined using a NanoDrop spectrophotometer (#NDL-PLUS-PR-CN, Invitrogen, Thermo Fisher Scientific, Carlsbad, CA, USA). Complementary DNA (cDNA) was synthesized using a reverse transcription kit (#11120ES60, Yeasen, Shanghai, China). qRT-PCR was performed using SYBR Green Master Mix (#11202ES08, Yeasen, Shanghai, China) on an ABI 7500 Real-Time PCR System (#4351105, Thermo Fisher Scientific, Carlsbad, CA, USA). The relative expression levels of target genes were calculated using the 2^–ΔΔCt^ method. The primers for qRT-PCR are listed in Table S1.

### 2.7 Macrophage Polarization

After seeding at a density of 1 × 10^6^ cells per well in 6-well plates, THP-1 cells were differentiated into adherent, resting macrophage-like cells termed THP-1(Mφ) by treatment with 100 ng/mL phorbol 12-myristate 13-acetate (PMA; #HY-18739, MCE, Monmouth Junction, NJ, USA). To prepare HCC-derived conditioned medium (CM), SDHA-knockdown Hep3B and SDHA-overexpressing MHCC97H cells were maintained in serum-free medium for 24 h. The collected medium was then centrifuged at 3000 rpm for 10 min using a Sorvall Legend Micro 17R centrifuge (#75002543, Invitrogen, Thermo Fisher Scientific, Carlsbad, CA, USA) and supplemented with 10% fetal bovine serum (#AUS-01S, Cellbox, Suzhou, China) and 1% penicillin–streptomycin (#PB180120, Pricella, Wuhan, China) to generate the final HCC-derived CM. Subsequently, THP-1(Mφ) cells were further treated with this HCC-derived CM or 1.5 mM succinate (#HY-N0420, MCE, Monmouth Junction, NJ, USA) for 48 h. For inhibition experiments, THP-1(Mφ) cells were pretreated for 6 h with 4 μM NF-56-EJ40 (#HY-130246, MCE, μ) and/or 5 μM Stattic (#HY-13818, MCE, Monmouth Junction, NJ, USA), followed by a 48 h co-incubation with 1.5 mM succinate in the continued presence of the inhibitors.

### 2.8 WB

Total protein was extracted from HCC cells (MHCC97H and Hep3B) and macrophages using RIPA reagent (#P0013B, Beyotime, Shanghai, China). After denaturation by high-temperature heating, proteins were separated by 10% SDS-PAGE and transferred to a nitrocellulose membrane (#66485, Pall, NY, USA). Membranes were blocked using 5% powdered milk (#LP0033B, Solarbio, Beijing, China) in Tris-buffered saline comprising Tween 20 (#T1085, Solarbio, Beijing, China) at room temperature for 1 h. Subsequently, the membrane was incubated with specific primary antibodies at room temperature for 2 h, including anti-SDHA (1:1000, #T56752, Abmart, Shanghai, China), anti-CD206 (1:1000, #ET1702-04, HUABIO, Hangzhou, China), anti-p-STAT3 (1:1000, #YP0251, Immunoway, Suzhou, China), anti-STAT3 (1:1000, #T55292, Abmart, Shanghai, China), anti-TOMM20 (1:1000, #WL0362, Wanleibio, Shenyang, China), anti-NDUFB8 (1:1000, #ET7108-25, HUABIO, Hangzhou, China), anti-ATP5A1 (1:1000, #ET1703-53, HUABIO, Hangzhou, China), anti-UQCRC1 (1:1000, #WLR114, Wanleibio, Shenyang, China), anti-COX IV (1:1000, #WL02203, Wanleibio, Shenyang, China), and β-actin (1:3000, #P60035M, Abmart, Shanghai, China). After washing, membranes were incubated with HRP-conjugated secondary antibodies (mouse: 1:3000, #M21001, rabbit: 1:3000, #M21002, Abmart, Shanghai, China) for 1 h at room temperature. Protein bands were visualized using enhanced chemiluminescence reagent (#SW134, Seven, Beijing, China), and images were acquired using a fluorescence/chemiluminescence imaging system (#JP-K600, JiaPeng Technology Co., Ltd., Shanghai, China). Quantitative analysis of the results was performed using ImageJ software (V1.54g, National Institutes of Health, NIH, Bethesda, MD, USA).

### 2.9 Immunohistochemistry (IHC)

Tissues from humans and mice were embedded in paraffin and sectioned. After baking, deparaffinization, and antigen retrieval, the tissue sections were blocked with hydrogen peroxide (#PV-9000, ZSGB-BIO, Beijing, China) for 20 min. They were then incubated with anti-SDHA (1:300, #T56752, Abmart, Shanghai, China) or anti-CD163 (1:3000, #ab182422, Abcam, Cambridge, UK) for 2 h, followed by incubation with the PV-9000 two-step immunohistochemistry detection kit (ZSGB-BIO, Beijing, China) for 20 min. Subsequently, the sections were stained with a 1:20 diluted DAB solution (20×, #ZLI-9017, ZSGB-BIO, Beijing, China) and counterstained with 0.5% hematoxylin (#BA-4041, Baso, Zhuhai, China) for 30 s, then mounted and examined under a microscope (Axioscope 5, ZEISS, Oberkochen, Germany). The assessment of the samples was completed using the IHC profiler plugin in ImageJ (V1.54g, National Institutes of Health, NIH, Bethesda, MD, USA).

### 2.10 Estimation of SDH Activity, Succinate, and Cytokine Concentrations

SDHA-knockdown Hep3B and SDHA-overexpressing MHCC97H cells were seeded in 96-well plates at a density of 1 × 10^4^ cells per well. After a 24h incubation, SDH activity was assessed using 0.5 mg/mL methyl thiazolyl tetrazolium (MTT, #HY-15924, MCE, Monmouth Junction, NJ, USA) for 4 h. Finally, the culture medium was discarded, and 100 μL of dimethyl sulfoxide (DMSO; #HY-Y0320C, MCE, Monmouth Junction, NJ, USA) was added to each well. The absorbance was measured at 570 nm using a microplate reader (#keebio-MR100, JiaPeng Technology Co., Ltd., Shanghai, China).

Paired HCC and adjacent normal tissues were homogenized in 0.9% physiological saline (#IN9000, Solarbio, Beijing, China). The tissue homogenates, serum, and CM from Hep3B and MHCC97H cells were centrifuged at 3000 rpm for 10 min using a Sorvall Legend Micro 17R centrifuge (#75002543, Invitrogen, Thermo Fisher Scientific, Carlsbad, CA, USA). The supernatants were collected and the succinate concentration was measured using a succinate assay kit (#MB-00547A, MBBiology, Nanjing, China). The absorbance was measured at 450 nm using a microplate reader (#keebio-MR100, JiaPeng Technology Co., Ltd., Shanghai, China).

Cytokines (tumor necrosis factor-alpha [TNF-α] and transforming growth factor-beta [TGF-β]) in macrophage-derived CM were measured with specific enzyme-linked immunosorbent assay (ELISA) kits, both from Invitrogen (TNF-α: #88-7346-88, TGF-β: # 88-8350-88, Thermo Fisher Scientific, Carlsbad, CA, USA). The absorbance was measured at 450 nm using a microplate reader (#keebio-MR100, JiaPeng Technology Co., Ltd., Shanghai, China).

### 2.11 Immunofluorescence Staining Assay and Flow Cytometry

After the designated interventions, macrophages were then fixed in 4% paraformaldehyde (#Sl101, Seven, Beijing, China) for 30 min and blocked with 10% goat serum (#SL038, Solarbio, Beijing, China) for 1 h. Following blocking, the cells were incubated with anti-CD206 (1:100, #ET1702-04, HUABIO, Hangzhou, China) and anti-CD68 (1:100, #HA601115, HUABIO, Hangzhou, China) for 2 h, followed by incubation with secondary antibodies (mouse: 1:500, #S7001, rabbit: 1:500, #S6002, Report, Shijiazhuang, China) for 1 h. Finally, the samples were sealed with mounting agent containing DAPI (#G1407, Servicebio, Wuhan, China) and visualized and photographed under a fluorescence microscope (Axioscope 5, ZEISS, Oberkochen, Germany).

After the designated interventions, macrophages were collected and washed with phosphate-buffered saline (PBS, pH = 7.2–7.4, 1×), followed by labeling with a FITC-CD206 (#MA5-16870, Invitrogen, CA, USA). The number of CD206^+^ cells was detected and quantified by CytoFLEX LX flow cytometry (Beckman Coulter, Suzhou, China).

### 2.12 Estimation of Mitochondrial DNA (mtDNA) Copy Number

mtDNA was extracted from macrophages using an extraction kit (#G3633, Servicebio, Wuhan, China) according to the manufacturer’s instructions. Subsequently, qPCR analysis was performed to determine the relative mtDNA copy number using a specific primer set targeting a conserved region of the mtDNA (Table S2) and normalized to a single-copy nuclear gene as an internal control.

### 2.13 Estimation of Reactive Oxygen Species (ROS), Mitochondrial Membrane Potential (MMP), Superoxide Dismutase (SOD), and Adenosine Triphosphate (ATP)

After the specified interventions, macrophages were collected and suspended in DCFH-DA solution (#S0033, Beyotime, Shanghai, China), followed by incubation at 37°C for 20 min. After incubation, the cells were washed twice with PBS (pH = 7.2–7.4, 1×) and resuspended in pre-cooled PBS (pH = 7.2–7.4, 1×). Cellular ROS levels were then measured by CytoFLEX LX flow cytometry (Beckman Coulter, Suzhou, China).

The THP-1(Mφ) cells were treated according to the experimental groups. Subsequently, after washing with PBS (pH = 7.2–7.4, 1×), the macrophages were incubated with JC-1 working solution (#C2006, Beyotime, Shanghai, China) for 20 min in a 37°C incubator. After two washes with JC-1 staining buffer, the cells were observed under a fluorescence microscope (Axioscope 5, ZEISS, Oberkochen, Germany).

Macrophages were lysed using RIPA reagent (#P0013B, Beyotime, Shanghai, China) and then centrifuged at 12,000× *g* for 5 min using a Sorvall Legend Micro 17R centrifuge (#75002543, Invitrogen, Thermo Fisher Scientific, Carlsbad, CA, USA). The protein concentration in the supernatant was determined using a BCA protein assay kit (#RW0201, Report, Shijiazhuang, China). According to the manufacturer’s instructions, WST-8/enzyme working solution and reaction initiation solution were prepared. Following the vendor’s guidelines (#S0101S, Beyotime, Shanghai, China), the supernatant was taken and mixed with the WST-8/enzyme working solution and reaction initiation solution. After incubation at 37°C for 30 min, the absorbance was measured at 450 nm using a microplate reader (#keebio-MR100, JiaPeng Technology Co., Ltd., Shanghai, China). SOD activity was calculated using a standard curve.

ATP levels in macrophages were determined using the ATP assay kit (#A095-1-1, Nanjing Jiancheng Bioengineering Institute, China). Briefly, cell pellets were collected by centrifugation and homogenized in 500 μL of ice-cold double-distilled water. After determining protein concentration using the BCA protein assay kit (#RW020, Report, Shijiazhuang, China), the remaining homogenate was boiled for 10 min and centrifuged at 3500 rpm for 10 min using a Sorvall Legend Micro 17R centrifuge (#75002543, Thermo Fisher Scientific, Carlsbad, CA, USA) to obtain the supernatant. The reaction system containing the sample, substrate solutions (I-IV), and chromogenic agent was incubated at 37°C for 30 min. The absorbance was measured at 636 nm using a microplate reader (#keebio-MR100, JiaPeng Technology Co., Ltd., Shanghai, China). ATP content was calculated using the following equation: $\text{C}=\left(\frac{A_{sample}-A_{control}}{A_{standard}-A_{blank}}\right)\times {\text{C}}_{\text{standard}}\times \text{dilution factor}/\text{protein concentration}$. In this equation, C is the sample ATP concentration; the A terms represent absorbance for the test, control, standard, and blank tubes; and C_standard_ is the known ATP standard concentration.

### 2.14 Proliferation and Cloning Formation Assay

The viability of HCC cells (MHCC97H and Hep3B) was evaluated using the Cell Counting Kit-8 (#C0038, Beyotime, Shanghai, China). HCC cells were seeded into 96-well plates at a density of 1 × 10^4^ cells per well and treated with CM from macrophages under different interventions. At various time points, CCK-8 reagent was added to each well and incubated for 1 h. The absorbance was measured at 450 nm using a microplate reader (#keebio-MR100, JiaPeng Technology Co., Ltd., Shanghai, China).

HCC cells (MHCC97H and Hep3B) were seeded at a density of 3000 cells per well in 6-well plates and continuously cultured with macrophage-CM for approximately 2 weeks to observe the number of cell colonies. Subsequently, the cells were fixed with 4% paraformaldehyde (#Sl101, Seven, Beijing, China) for 30 min and stained with 1% crystal violet (#G1062, Solarbio, Beijing, China) for 20 min. After imaging, the colonies were counted using ImageJ software (V1.54g, National Institutes of Health, NIH, Bethesda, MD, USA).

### 2.15 Wound-Healing and Transwell Invasion Assay

HCC cells (MHCC97H and Hep3B) were seeded into 6-well plates at a density of 3 × 10^5^ cells per well. When the cells reached approximately 90% confluence, a scratch was made across the monolayer using a pipette tip and floating cells were removed by washing with PBS (pH = 7.2–7.4, 1×). The cells were then cultured in macrophage-CM for 24 h. The degree of wound healing was assessed by comparing the scratch width at 24 h with that at 0 h.

The invasive capacity of HCC cells (MHCC97H and Hep3B) was assessed using Matrigel transwell inserts (#354480, Corning, NY, USA). Cells were seeded into the upper chamber, while macrophage-CM was added to the lower chamber. Following incubation for 24 h, cells were fixed with 4% paraformaldehyde (#Sl101, Seven, Beijing, China) for 30 min and stained with 1% crystal violet (#G1062, Solarbio, Beijing, China) for 20 min. Finally, images were captured under a microscope (DMIL LED, Leica, Wetzlar, Germany), and the number of invasive cells was analyzed using ImageJ software (V1.54g, National Institutes of Health, NIH, Bethesda, MD, USA).

### 2.16 Mouse Models

The animal experiments were approved by the Experimental Animal Ethics Committee of the Hebei Medical University Third Hospital (No. Z2024-036-2). This ensures that the study adheres to the national and international guidelines for the care and use of laboratory animals. All methods were performed according to the relevant guidelines and regulations.

A total of 18 male C57BL/6J mice (6 weeks old, weighing about 20 g) were purchased from Zizhen Biotechnology Company (Hebei, China). All mice were housed under specific pathogen-free conditions at a constant temperature of 23°C with a 12-h light/dark cycle, and had ad libitum access to sterilized water and standard laboratory diet. In the tumor growth experiments, 5 × 10^6^ Hepa1-6 cells suspended in 100 μL of PBS (pH = 7.2–7.4, 1×) were subcutaneously injected into the back of C57BL/6J mice. When the average tumor volume reached approximately 100 mm^3^, the mice were randomly assigned into three groups (*n* = 6 per group) and treated via intraperitoneal injection twice weekly. The control group was administered an equal volume of PBS (pH = 7.2–7.4, 1×); the succinate group received 100 mg/kg succinate; and the inhibitor group was co-administered 100 mg/kg succinate and 100 mg/kg of the GPR91 inhibitor (compound 4c, #HY-126217, MCE, Monmouth Junction, NJ, USA). During the treatment period, tumor size and pain/distress classifications were monitored every 3 days. Tumor volume (mm^3^) was calculated using the following equation: V = $\left(\frac{\text{length}(\text{L})\times \text{width}(\text{W}{)}^{2}}{2}\right)$. In this equation, L and W represent the tumor’s longest longitudinal diameter (length) and shortest transverse diameter (width), respectively. After euthanasia, tumor samples were weighed and stored for further analysis. Longitudinal blood sampling was performed through tail vein puncture at pre-intervention baseline and study termination timepoints.

### 2.17 Statistical Analysis

All statistical analyses were performed using the GraphPad Prism software (10.1.2, GraphPad Software, San Diego, CA, USA). Data are presented as the mean ± standard deviation (SD) or standard error of the mean (SEM). The diagnostic efficacy of succinate for predicting HCC progression was evaluated by employing area under the receiver operating characteristic curve analysis. Statistical differences between two groups were evaluated using the one-tailed unpaired or paired Student’s *t*-test. Correlation analyses were performed using Pearson’s rank correlation test. *p*-value < 0.05 was considered statistically significant.

## 3 Results

### 3.1 Higher Proportion of M2 Macrophages in Tumor Tissues Correlates with Poor Prognosis of HCC Patients

**Figure 1:**
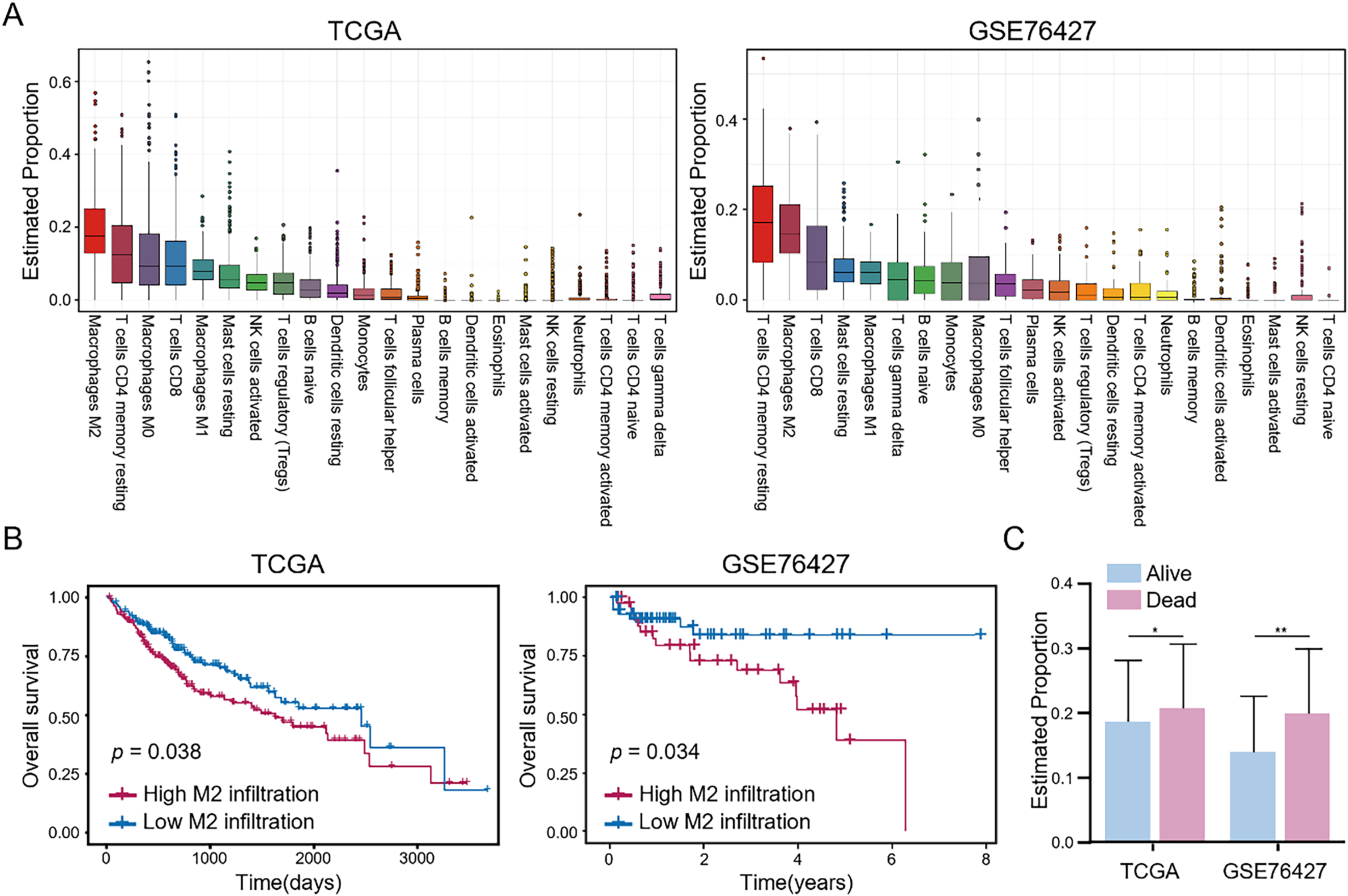
Evaluation of immune cell infiltration and its impact on the prognosis of HCC patients. (**A**) Infiltration levels of 22 immune cell types in HCC tissues were evaluated by CIBERSORT and presented from high to low. (**B**) Impact of M2 macrophage infiltration levels on the overall survival of HCC patients. (**C**) Comparison of M2 macrophage infiltration levels between the survival and deceased groups in HCC patients. Data are presented as mean ± SD. *p < 0.05, **p < 0.01

We initially assessed the infiltration status of 22 immune cell types in HCC samples from the TCGA and GSE76427 cohorts. As shown in Fig. 1A, M2 macrophages and resting CD4^+^ memory T cells exhibited the highest relative abundance among the 22 infiltrating immune cell types. Subsequently, we analyzed the relationship between the infiltration levels of these two types of immune cells and the prognosis of HCC patients. As shown in Figs. 1B and S1, HCC patients with high M2 macrophage infiltration showed significantly lower overall survival (OS) compared to those with low M2 macrophage infiltration; moreover, patients with high resting CD4^+^ memory T cell infiltration showed slightly higher OS than those with low infiltration, although the difference was not statistically significant. Furthermore, M2 macrophage infiltration levels were significantly higher in the deceased group than in the survival group across both HCC cohorts (Fig. 1C). These findings suggest that higher infiltration of M2 macrophages in tumor tissues correlates significantly with a worse prognosis of HCC patients.

### 3.2 SDHA is a Key Metabolism-Related DEG Associated with M2 Macrophages

**Figure 2:**
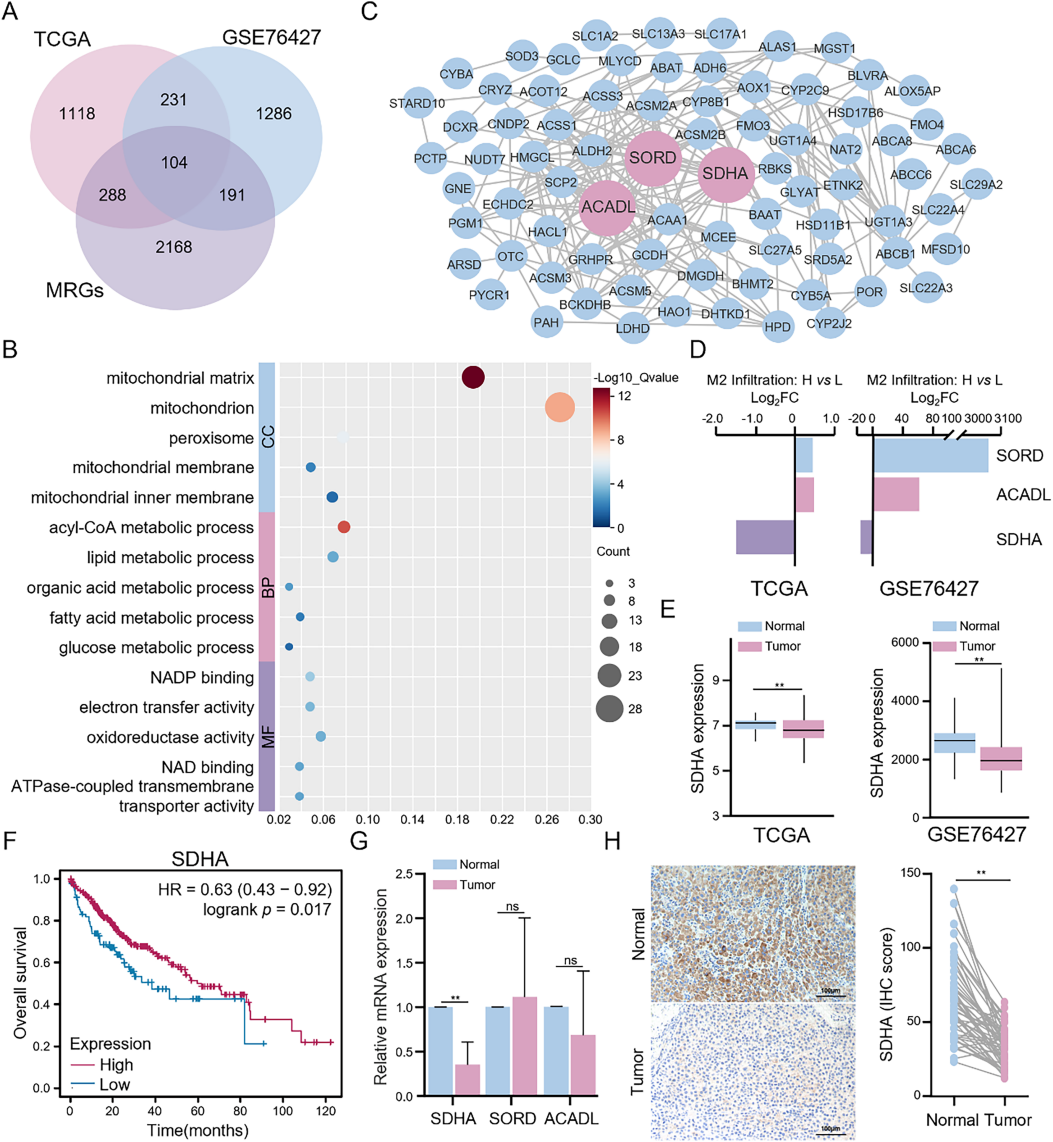
Identification and validation of key metabolism-related DEGs. (**A**) Intersection analysis of DEGs between high and low M2 macrophage infiltration groups and metabolism-related genes in HCC. (**B**) Functional enrichment analysis of metabolism-related DEGs was conducted through the DAVID database. (**C**) PPI network of metabolism-related DEGs was constructed based on the STRING database. (**D**) Differential expression of three key metabolism-related DEGs (ACADL, SORD, and SDHA). (**E**) Comparison of SDHA expression between HCC and normal tissues in the TCGA cohort from the TIMER 2.0 database and the GSE76427 cohort. (**F**) Impact of SDHA mRNA levels on the overall survival of HCC patients from the Kaplan-Meier plotter database. (**G**) Results of qRT-PCR for determining the relative mRNA expression of ACADL, SORD, and SDHA between tumor and paired normal tissues in real-world HCC patients (*n* = 17). (**H**) Representative IHC images of SDHA expression in real-world HCC tumors and paired normal tissues (*n* = 52). Data are presented as mean ± SD. DEGs, differentially expressed genes; MRGs: metabolism-related genes; CC, cellular component; BP, biological process; MF, molecular function; FC, fold change. **p < 0.01, ns, not significant

To elucidate the mechanisms regulating the infiltration of M2 macrophages in the TME of HCC tissues, we analyzed the gene expression data from the high and low M2 macrophage infiltration groups in the TCGA and GSE76427 cohorts. Transcriptome analysis identified 1741 and 1812 DEGs in the TCGA and GSE76427 cohorts, respectively. Given that the intricate crosstalk between tumor cells and immune cells is associated with the metabolic processes, we identified 104 metabolism-related DEGs among the overall DEGs (Fig. 2A). Functional enrichment analysis of these metabolism-related DEGs revealed that they were primarily associated with mitochondrial functions, including energy metabolism, redox reactions, and transmembrane transport (Fig. 2B). PPI network analysis demonstrated that long-chain acyl-coA dehydrogenase (ACADL), sorbitol dehydrogenase (SORD), and SDHA were the three metabolism-related genes with the highest degree of connectivity (Fig. 2C). Additionally, higher infiltration of M2 macrophages in HCC tissues was associated with the increased expression of ACADL and SORD and decreased expression of SDHA (Fig. 2D). Gene expression analysis revealed that the expression levels of ACADL, SORD, and SDHA were lower in HCC tissues than in adjacent normal tissues in both TCGA and GSE76427 cohorts (Figs. 2E and S2A). Survival analysis indicated that the low expression levels of ACADL, SORD, and SDHA were significantly associated with worse prognosis (Figs. 2F and S2B). Next, we analyzed the expression levels of these 3 DEGs in 17 paired HCC and adjacent normal tissues. qRT-PCR analysis demonstrated that SDHA expression was significantly downregulated in HCC tissues compared to that in adjacent normal liver tissues (Fig. 2G). The expression levels of ACADL and SORD showed no significant difference between HCC and adjacent normal tissues. IHC confirmed that SDHA expression was reduced in HCC tissues (Fig. 2H). These findings indicate that SDHA is a crucial metabolism-related DEG in HCC and is closely associated with higher infiltration of M2 macrophages into the tumor tissues.

### 3.3 SDHA Deficiency in HCC Promotes M2 Macrophage Polarization

**Figure 3:**
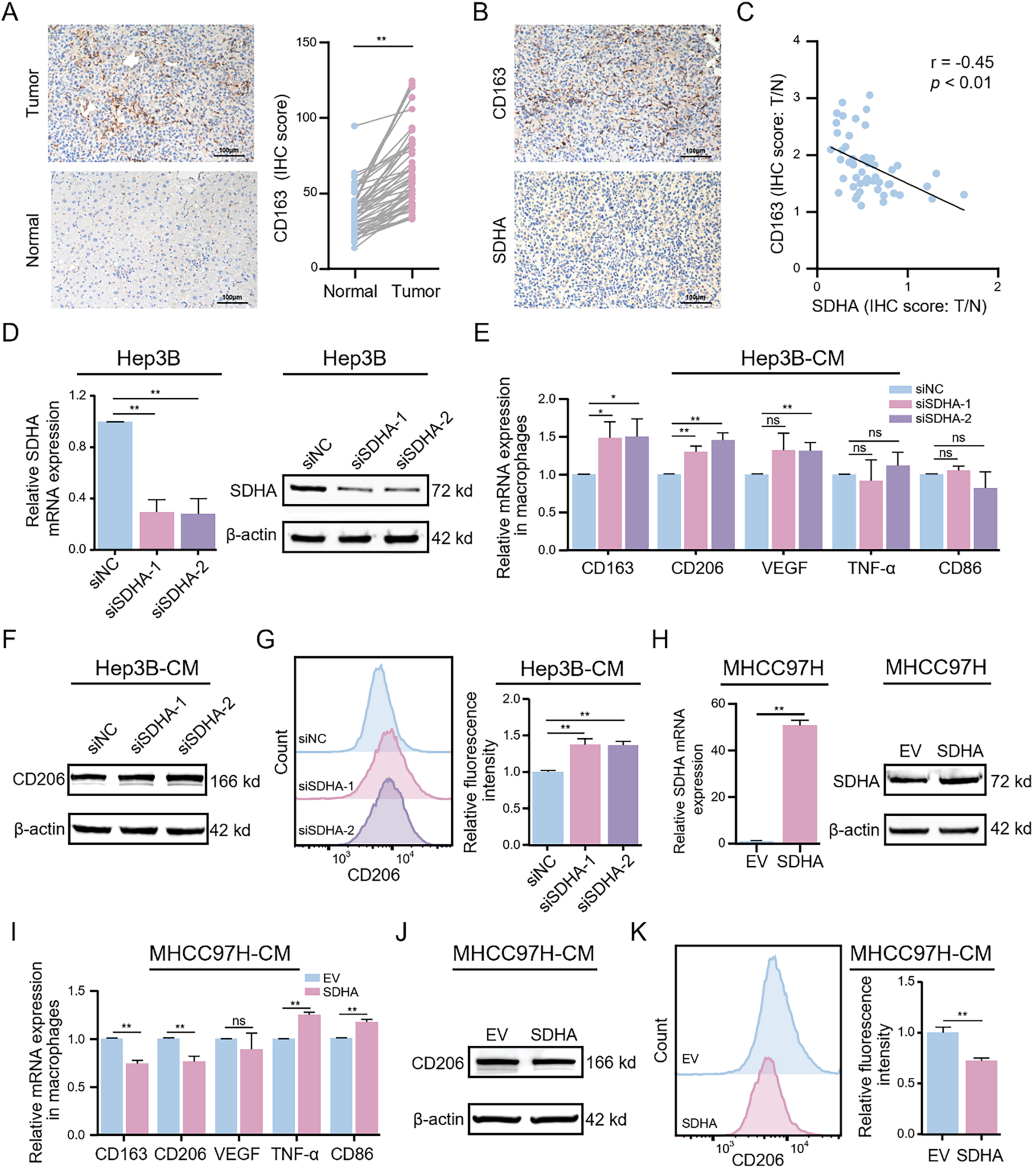
Genetic alterations in HCC-associated SDHA regulate M2 macrophage polarization. (**A**) Representative IHC images of CD163 expression in real-world HCC tumors and paired normal tissues (*n* = 52). (**B**) Representative IHC images of SDHA and CD163 in paired HCC tumor tissues (*n* = 52). (**C**) Correlation coefficient between SDHA and CD163 in HCC based on the IHC score ratio (*n* = 52, T/N). (**D**) Changes in SDHA mRNA and protein expression levels after SDHA knockdown in Hep3B cells were detected by qRT-PCR and WB assay. THP-1(Mφ) cells were treated with CM from SDHA-knockdown Hep3B cells. (**E**) Relative mRNA expression of M1 or M2 markers was analyzed by qRT-PCR. (**F**) Protein expression level of CD206 was detected by WB assay. (**G**) Percentage changes of CD206^+^ macrophages were analyzed by flow cytometry. (**H**) Changes in SDHA mRNA and protein expression levels after SDHA overexpression in MHCC97H cells were detected by qRT-PCR and WB assay. THP-1(Mφ) cells were treated with CM from SDHA-overexpressing MHCC97H cells. (**I**) Relative mRNA expression of M1 or M2 markers was analyzed by qRT-PCR. (**J**) Changes in CD206 protein expression levels were detected by WB assay. (**K**) Percentage changes of CD206^+^ macrophages were analyzed by flow cytometry. Data are presented as mean ± SD. T, tumor. N, normal. THP-1(Mφ), macrophages induced from THP-1 cells. CM, conditioned medium. *p < 0.05, **p < 0.01, ns, not significant

We further investigated the potential association between SDHA and M2 macrophages. Initially, we used IHC to assess the infiltration of M2 macrophages (cluster of differentiation 163 [CD163]) in HCC tissues. Compared to adjacent normal tissues, HCC tumor tissues showed significantly elevated expression levels of CD163 (Fig. 3A). Correlation analysis demonstrated a significant negative correlation between SDHA and CD163 expression levels in HCC tumor tissues (r = −0.45, *p* < 0.01) (Fig. 3B,C). To further elucidate the role of SDHA in the infiltration of macrophages in HCC tissues, SDHA was knocked down or overexpressed in HCC cells. Subsequently, the CM of these cells was used for co-culturing with THP-1(Mφ). The expression levels of SDHA were analyzed in multiple HCC cell lines. As shown in Fig. S3, compared to THLE-2 cells, HCC cell lines exhibited a significant decrease in the mRNA level of SDHA. Subsequently, we established Hep3B cells with significant knockdown of SDHA (Fig. 3D). Stimulation of THP-1(Mφ) cells with CM from SDHA-knockdown HCC cells significantly increased the mRNA expression levels of M2 macrophage markers such as cluster of differentiation 206 (CD206), CD163, and vascular endothelial growth factor (VEGF) (Fig. 3E). WB assay (Fig. 3F) and flow cytometry analysis (Fig. 3G) of these stimulated THP-1(Mφ) cells demonstrated increased expression of CD206 and an elevated number of CD206^+^ cells, respectively. This finding suggests that SDHA knockdown promoted M2 macrophage polarization. We also generated stably SDHA-overexpressing MHCC97H cells (Fig. 3H). Stimulation of THP-1(Mφ) cells with CM from SDHA-overexpressing MHCC97H cells showed a significant decrease in the mRNA expression of CD206 and CD163 and a significant increase in the mRNA expression of M1 macrophage markers: TNF-α and cluster of differentiation 86 (CD86) (Fig. 3I). WB assay (Fig. 3J) and flow cytometry analysis (Fig. 3K) demonstrated that stimulation of THP-1(Mφ) cells with CM from SDHA-overexpressing MHCC97H cells decreased CD206 expression and the number of CD206^+^ cells, respectively, compared to the corresponding controls. This observation suggests that SDHA overexpression in HCC cells inhibited the polarization of M2 macrophages. These findings indicate that SDHA plays a crucial role in regulating M2 macrophage polarization. Therefore, altered expression levels of SDHA in HCC cells can significantly influence macrophage polarization states.

### 3.4 SDHA Deficiency-Induced Succinate Accumulation Promotes M2 Polarization of Macrophages

**Figure 4:**
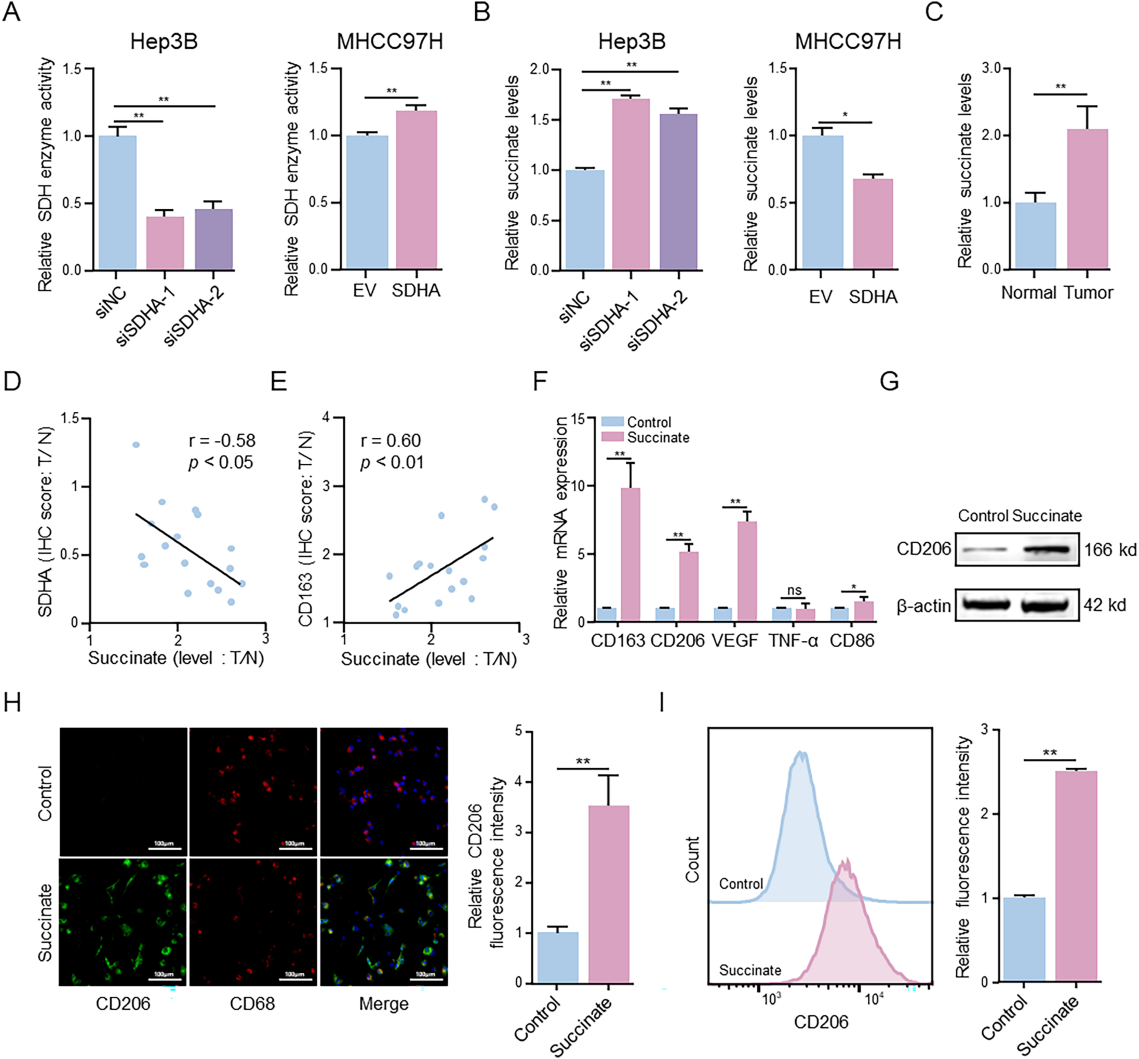
Succinate induces macrophage M2 polarization. (**A**) MTT assay detected alterations in SDH activity in SDHA-knockdown Hep3B cells and SDHA-overexpression MHCC97H cells. (**B**) ELISA evaluated changes in succinate concentration in SDHA-knockdown Hep3B cells and SDHA-overexpression MHCC97H cells. (**C**) ELISA measured the concentration of succinate in real-world HCC tissues and paired normal tissues. (**D**,**E**) Correlation coefficient between succinate concentration and SDHA and CD163 in paired HCC tissues based on the T/N ratio (*n* = 17). THP-1(Mφ) cells were treated with or without succinate (1.5 mM) for 48 h. (**F**) Relative mRNA expression of M1 or M2 markers was analyzed by qRT-PCR. (**G**) The protein level was detected by WB assay. (**H**) Representative immunofluorescence images of CD206 (green) and CD68 (red) co-staining. Cell nuclei were counterstained with DAPI. (**I**) Percentage changes of CD206^+^ macrophages were analyzed by flow cytometry. Data are presented as mean ± SD. T, tumor; N, normal; THP-1(Mφ), macrophages induced from THP-1 cells. *p < 0.05, **p < 0.01, ns, not significant

SDHA functions as the core catalytic subunit of the SDH enzyme complex and catalyzes the oxidation of succinate to fumarate. To elucidate the role of SDHA in SDH activity, we evaluated the effects of SDHA knockdown or overexpression on SDH activity. Compared to the control group, SDHA knockdown significantly reduced SDH activity, while SDHA overexpression enhanced SDH activity in HCC cells (Fig. 4A). Next, we evaluated changes in succinate levels following SDHA knockdown or overexpression. Compared to the control group, succinate levels were significantly higher in SDHA-knockdown HCC cells and were significantly reduced in SDHA-overexpressing HCC cells (Fig. 4B). To investigate the potential role of succinate in HCC, we estimated succinate levels in HCC and adjacent normal liver tissues. Succinate levels were significantly higher in HCC tissues than in adjacent normal liver tissues (Fig. 4C). We also analyzed the correlation between succinate levels and the expression levels of SDHA and CD163 in HCC tissues. Our data showed a significant negative correlation between succinate levels and SDHA expression (r = −0.58, *p* < 0.05) (Fig. 4D) and a significant positive correlation between succinate levels and CD163 expression (r = 0.60, *p* < 0.01) (Fig. 4E) in HCC tissues. These findings indicate that succinate promotes HCC progression by modulating M2 macrophage polarization.

To determine the regulatory role of succinate in M2 macrophage polarization, THP-1(Mφ) cells were treated with succinate. qRT-PCR analysis (Fig. 4F) demonstrated that succinate treatment significantly upregulated the mRNA levels of CD163, CD206, and VEGF. WB assay (Fig. 4G) and immunofluorescence assay (Fig. 4H) further demonstrated that succinate treatment significantly increased CD206 protein expression in the succinate-treated group compared to that in the control group. Flow cytometry analysis (Fig. 4I) indicated a significantly higher proportion of CD206^+^ cells in the succinate-treatment group than in the control group. Additionally, ELISA (Fig. S4A,B) showed significantly decreased TNF-α levels and increased TGF-β levels in the succinate-treated group compared to those in the control group. Collectively, these results suggest that SDHA deficiency leads to succinate accumulation, which subsequently promotes M2 polarization during HCC progression.

### 3.5 GPR91 Mediates Succinate-Induced M2 Macrophage Polarization

**Figure 5:**
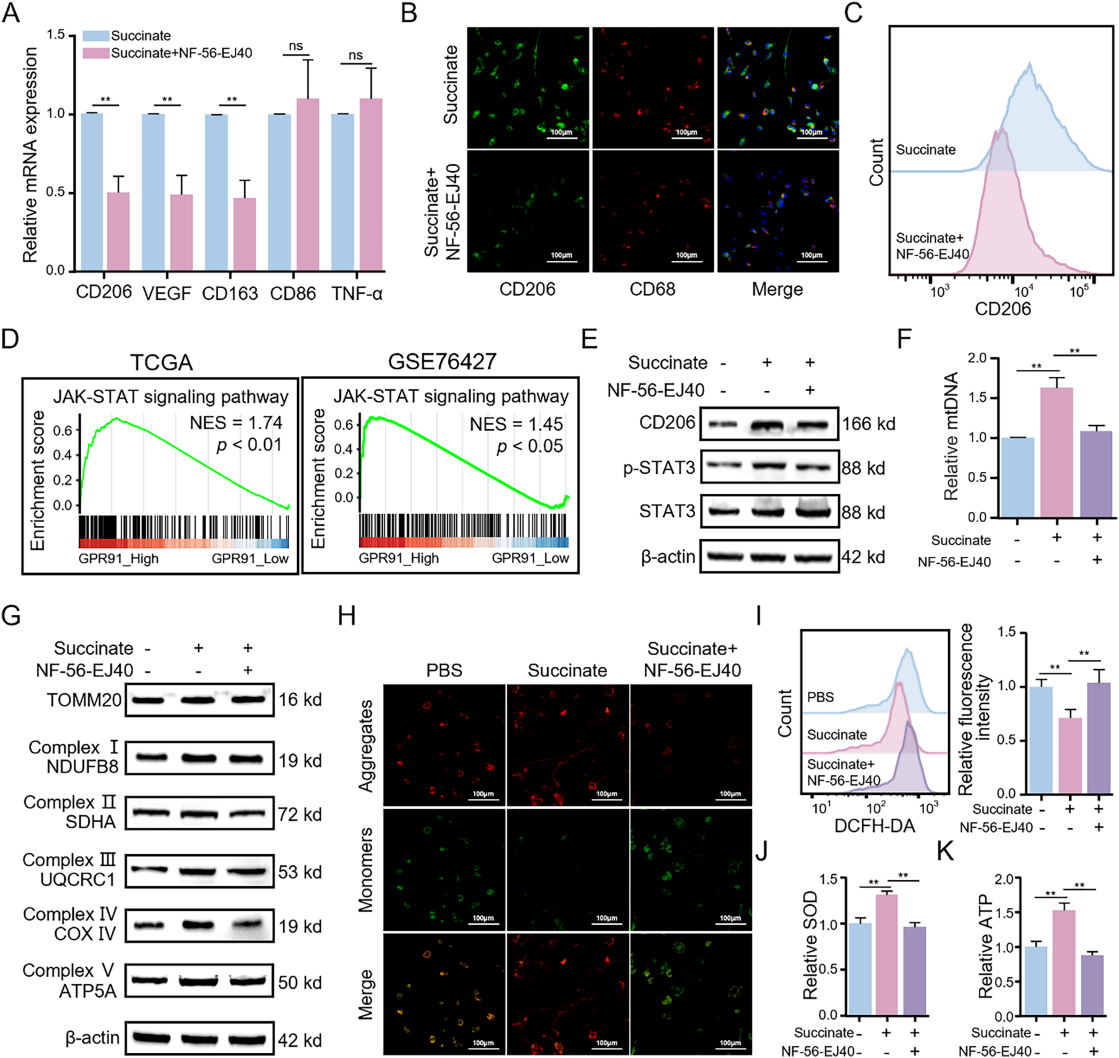
Succinate-induced M2 macrophage polarization depends on the membrane receptor GPR91 and enhances mitochondrial function. THP-1(Mφ) cells were pretreated with or without the GPR91 inhibitor NF-56-EJ40 (4 μM) for 6 h, followed by exposure to succinate (1.5 mM) for 48 h. (**A**) Relative mRNA expression of M1 or M2 markers was detected by qRT-PCR. (**B**) Representative immunofluorescence images of co-staining for CD206 (green) and CD68 (red). Cell nuclei were counterstained with DAPI. (**C**) Percentage changes of CD206^+^ macrophages were analyzed by flow cytometry. (**D**) GSEA revealed that GPR91 expression is positively correlated with the JAK-STAT3 signaling pathway in TCGA and GSE76427 datasets. (**E**) Protein levels of CD206 and p-STAT3 were detected by WB assay. (**F**) Relative expression of mtDNA was analyzed by qRT-PCR. (**G**) Protein levels of TOMM20 and mitochondrial complex proteins (NDUFB8, SDHA, UQCRC1, COX IV, and ATP5A) were detected by WB assay. (**H**) MMP was detected using the JC-1 probe. (**I**) ROS levels were measured by flow cytometry. (**J**) SOD levels were estimated using the SOD assay kit. (**K**) ATP levels were quantified using the ATP assay kit. Data are presented as mean ± SD. THP-1(Mφ), macrophages induced from THP-1 cells. **p < 0.01, ns, not significant

Previous studies have shown that exogenous succinate binds to GPR91 (also known as succinate receptor 1 [SUCNR1]), a cell surface receptor, and activates downstream signaling pathways [19]. We therefore investigated whether GPR91 mediates succinate-induced M2 macrophage polarization. THP-1(Mφ) cells were pretreated with the GPR91-specific inhibitor NF-56-EJ40 before stimulation with succinate (Fig. S5A). The results revealed that pretreatment with NF-56-EJ40 significantly reduced the mRNA levels of CD206, CD163, and VEGF after succinate treatment compared to those in the control group (Fig. 5A). ELISA showed that NF-56-EJ40 treatment significantly increased TNF-α level, while reducing TGF-β level (Fig. S5B,C). Immunofluorescence and flow cytometry analyses demonstrated that CD206 expression was significantly reduced in the NF-56-EJ40-treated group (Figs. 5B and S5D), with a lower proportion of CD206^+^ cells (Figs. 5C and S5E), respectively. These results were further corroborated by the WB assay (Fig. 5E).

To examine the functional role and molecular mechanisms of GPR91, we performed GSEA of the expression profiles from both TCGA and GSE76427 datasets based on high or low GPR91 expression levels. GSEA results revealed that HCC samples with high GPR91 expression were significantly enriched in janus kinase (JAK)-signal transducer and activator of transcription (STAT) signaling-related pathways (Fig. 5D). Because the STAT3 signaling pathway plays a crucial role in M2 macrophage polarization and functions downstream of GPR91 [20,21], we conducted an in-depth study of its involvement in this process. WB assay (Fig. 5E) showed that p-STAT3 levels in macrophages were significantly elevated after succinate treatment compared to those in the control group; however, this effect was reversed by pretreatment with NF-56-EJ40. These findings suggest that succinate-induced M2 macrophage polarization occurs through GPR91-mediated activation of the STAT3 signaling pathway.

### 3.6 Mitochondrial OXPHOS is Elevated in the Succinate-Induced M2 Macrophages

Mitochondrial OXPHOS has a key role in M2 macrophage polarization and regulates mitochondrial dynamics and homeostasis [22]. To examine this relationship, we investigated mitochondrial alterations in succinate-treated macrophages with or without NF-56-EJ40 pretreatment. We first analyzed changes in mtDNA copy number. Succinate treatment significantly elevated the relative mtDNA copy number in M2 macrophages compared to that in the control group; however, this was significantly reduced by NF-56-EJ40 pretreatment (Fig. 5F). WB assay further corroborated these findings. The expression levels of the mitochondrial marker protein translocase of outer mitochondrial membrane 20 (TOMM20) were significantly higher in succinate-treated M2 macrophages, but decreased in cells pretreated with NF-56-EJ40 (Fig. 5G). This finding suggests that succinate induced mitochondrial biogenesis in the M2 macrophages. Moreover, our analysis demonstrated significant upregulation of the expression levels of marker proteins from the mitochondrial respiratory chain complexes I-V following succinate treatment; however, this effect was neutralized by NF-56-EJ40 pretreatment (Fig. 5G). We also performed JC-1 staining to evaluate changes in MMP. MMP was significantly increased in macrophages following succinate treatment; however, pretreatment with NF-56-EJ40 decreased MMP (Fig. 5H). We also analyzed the effects of succinate on the intracellular ROS levels during M2 macrophage polarization. Succinate treatment decreased intracellular ROS levels in M2-polarized macrophages; however, NF-56-EJ40 pretreatment resulted in elevated intracellular ROS levels in succinate-treated macrophages (Fig. 5I). Similarly, SOD and ATP levels increased in succinate-treated macrophages but were decreased following NF-56-EJ40 pretreatment (Fig. 5J,K). These findings suggest that mitochondrial OXPHOS is significantly enhanced in succinate-induced M2 macrophages.

### 3.7 Succinate Induces M2 Macrophage Polarization by Activating the GPR91/STAT3 Signaling Pathway

**Figure 6:**
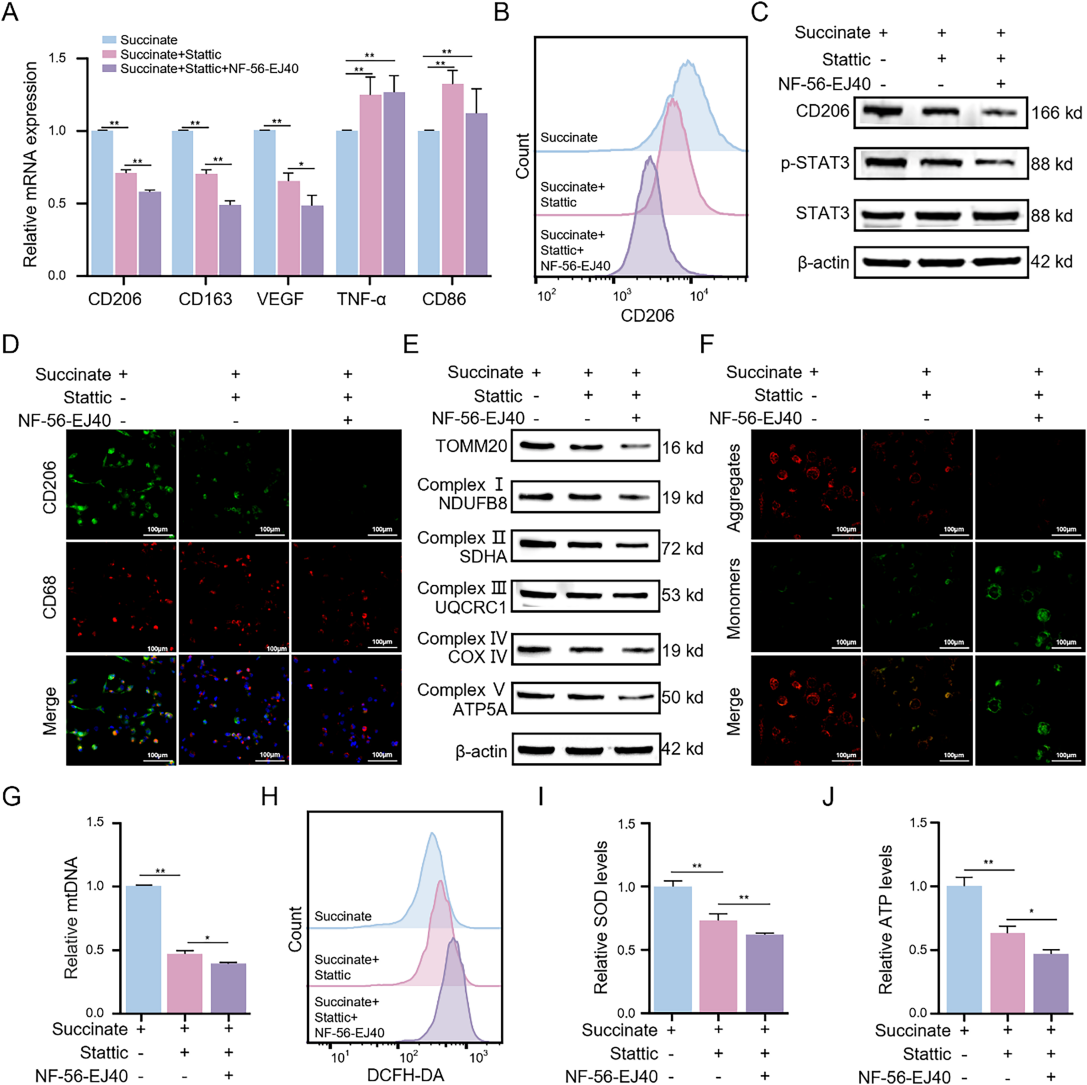
Succinate induces M2 macrophage polarization and enhances mitochondrial function through GPR91/STAT3 activation. THP-1(Mφ) cells were pretreated with the STAT3 inhibitor Stattic (5 μM) alone or in combination with the GPR91 inhibitor NF-56-EJ40 (4 μM) for 6 h, followed by exposure to succinate (1.5 mM) for 48 h. (**A**) Relative mRNA expression of M1 or M2 markers was detected by qRT-PCR. (**B**) Percentage change of CD206^+^ macrophages was analyzed by flow cytometry. (**C**) Protein levels of CD206 and p-STAT3 were estimated by WB assay. (**D**) Representative immunofluorescence images of co-staining for CD206 (green) and CD68 (red). Cell nuclei were counterstained with DAPI. (**E**) Protein expression levels of TOMM20 and mitochondrial complex proteins (NDUFB8, SDHA, UQCRC1, COX IV, and ATP5A) were quantified by WB assay. (**F**) MMP was detected using the JC-1 probe. (**G**) Relative expression of mtDNA was determined by qRT-PCR. (**H**) ROS levels were quantified by flow cytometry. (**I**) SOD levels were measured using the SOD assay kit. (**J**) ATP levels were estimated using the ATP assay kit. Data are presented as mean ± SD. THP-1(Mφ), macrophages induced from THP-1 cells. *p < 0.05, **p < 0.01

Next, we investigated whether GPR91 modulated succinate-induced M2 macrophage polarization through the STAT3 signaling cascade using the STAT3 inhibitor Stattic. qRT-PCR analysis (Fig. 6A) revealed that Stattic significantly reduced succinate-induced upregulation of CD206, CD163, and VEGF transcripts, but increased the transcript levels of TNF-α and CD86, which are markers of M1 macrophage phenotype. These effects were further enhanced by the combined treatment of macrophages with Stattic and NF-56-EJ40. Flow cytometric analysis (Figs. 6B and S6A) demonstrated that Stattic treatment reduced the percentage of CD206^+^ cells, whereas combined treatment of Stattic and NF-56-EJ40 further amplified the inhibitory effects. WB assay (Fig. 6C) and immunofluorescence assay (Figs. 6D and S6B) corroborated these findings and indicated that Stattic treatment significantly decreased the protein expression levels of CD206. Combined treatment with Stattic and NF-56-EJ40 further reduced the expression of the CD206 protein. Furthermore, ELISA (Fig. S6C,D) confirmed that Stattic treatment significantly elevated TNF-α while reducing TGF-β. Combined treatment with Stattic and NF-56-EJ40 markedly amplified these alterations. Mechanistically, combined treatment with NF-56-EJ40 and Stattic significantly downregulated the p-STAT3 protein level (Fig. 6C). Collectively, these results confirmed that succinate promotes M2 macrophage polarization by activating the GPR91/STAT3 signaling pathway.

### 3.8 GPR91/STAT3 Suppression Promotes Mitochondrial Dysfunction in Succinate-Induced M2 Macrophages

Given that mitochondrial OXPHOS was augmented in succinate-induced M2 macrophages, we evaluated the effects of Stattic and NF-56-EJ40 on mitochondrial function. WB assay showed that treatment with Stattic significantly reduced succinate-induced upregulation of TOMM20 and mitochondrial respiratory chain complexes I–V; these effects were further enhanced by combining Stattic with NF-56-EJ40 (Fig. 6E). Fluorescence imaging results further demonstrated that treatment with Stattic decreased MMP, and this effect was further amplified through combined treatment with Stattic and NF-56-EJ40 (Figs. 6F and S6E). Additionally, Stattic treatment significantly attenuated succinate-induced increase in mtDNA copy number, and NF-56-EJ40 treatment further augmented this effect (Fig. 6G). Stattic treatment also increased the ROS levels and reduced SOD and ATP levels in succinate-induced macrophages; these effects were further augmented by combined treatment with Stattic and NF-56-EJ40 (Fig. 6H–J). These findings demonstrate that GPR91/STAT3 pathway inhibition induces mitochondrial dysfunction and increases intracellular ROS levels in succinate-induced M2 macrophages.

### 3.9 Succinate-Induced M2 Macrophages Promote HCC Cell Proliferation, Migration, and Invasion In Vitro

**Figure 7:**
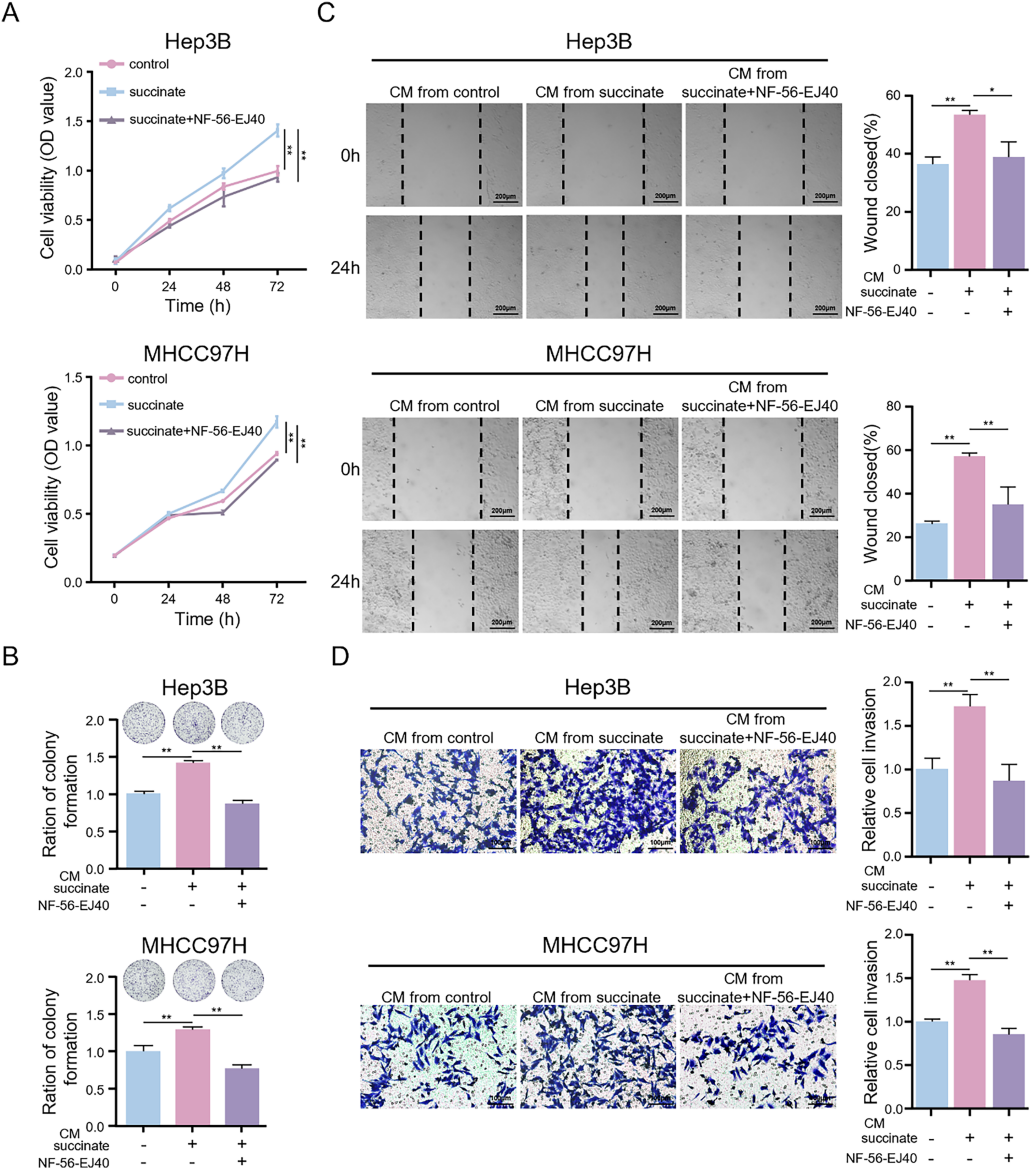
Succinate-induced M2 macrophages promote HCC cell proliferation, migration, and invasion *in vitro*. HCC cells were treated with the CM from THP-1(Mφ) cells pretreated with or without NF-56-EJ40 (4 μM) for 6 h and subsequently stimulated with succinate or PBS (pH = 7.2–7.4, 1×). (**A**,**B**) CCK-8 assay and colony formation assay to detect the proliferation ability of HCC cells. (**C**) Wound healing assay to evaluate the migration ability of HCC cells. (**D**) Transwell assay to examine the invasion ability of HCC cells. Data are presented as mean ± SD. CM, conditioned medium. *p < 0.05, **p < 0.01

To investigate the biological effects of succinate-induced M2 macrophages on HCC progression, we treated HCC cells with CM from macrophages treated with succinate with or without NF-56-EJ40 pretreatment. Compared to CM from untreated macrophages, CM derived from succinate-stimulated macrophages significantly increased HCC cell proliferation (Fig. 7A,B), migration (Fig. 7C), and invasiveness (Fig. 7D). Additionally, CM from macrophages treated with NF-56-EJ40 significantly reduced HCC cell proliferation, migration, and invasiveness compared to CM from macrophages treated with succinate alone. These findings indicate that succinate-induced M2 macrophages promote HCC cell growth and progression through a GPR91-dependent mechanism, which can be inhibited by GPR91 blockade.

### 3.10 Succinate-Induced M2 Macrophages Promote HCC Progression In Vivo

**Figure 8:**
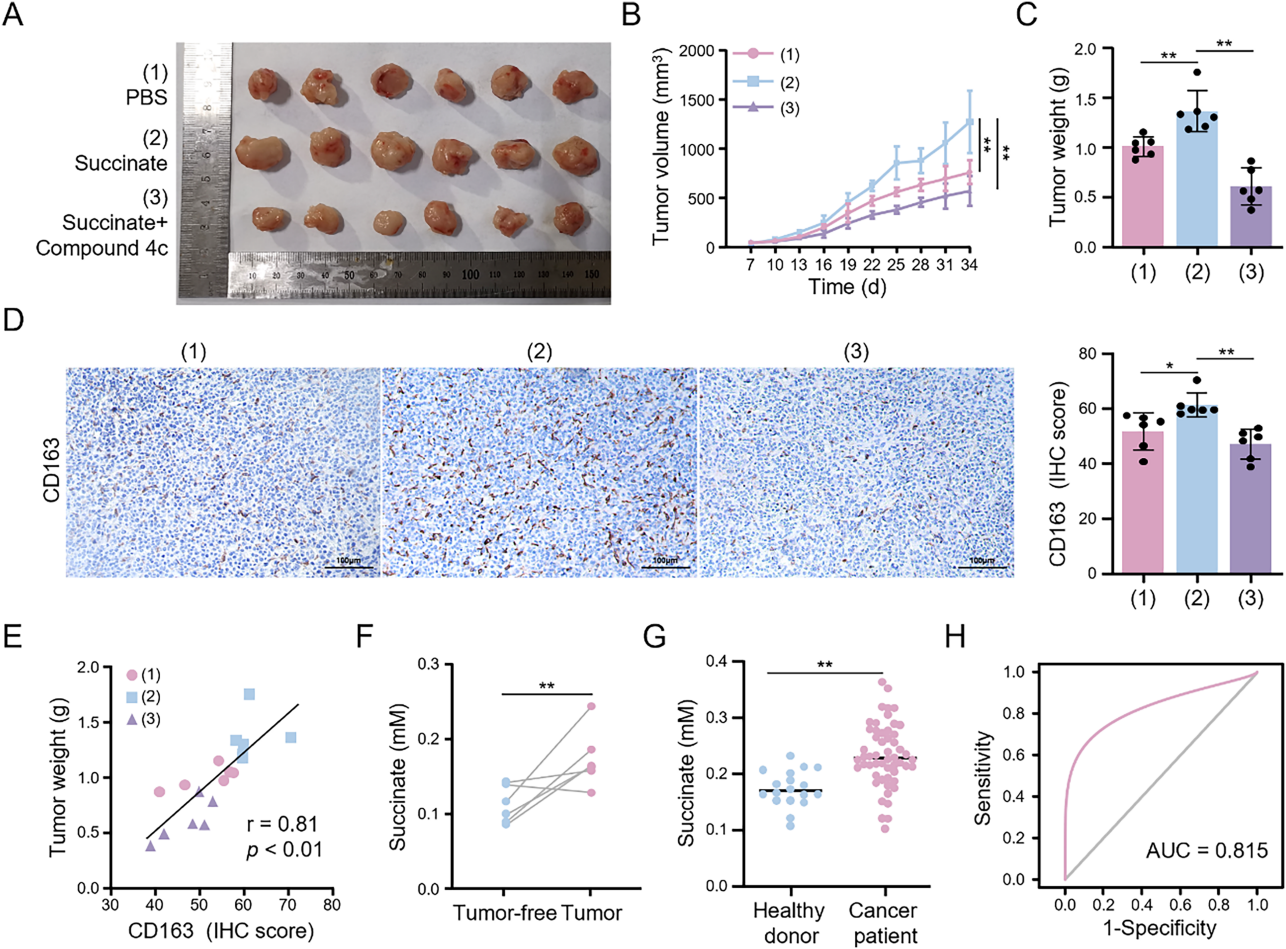
Succinate induces M2 macrophage infiltration to facilitate HCC progression and serves as a serum biomarker. Mice bearing subcutaneous tumors were randomly assigned to treatment with PBS (pH = 7.2–7.4, 1×), succinate, or succinate plus the GPR91 inhibitor (compound 4c) for 4 weeks. (**A**) Representative images showing tumors harvested from tumor-bearing tumors. (**B**) Quantitative analysis of tumor size. (**C**) Quantitative analysis of tumor weight. (**D**) Representative IHC images of CD163 expression in the tumors. (**E**) Correlation coefficient between tumor weight and CD163 expression. (**F**) Serum succinate concentrations in mice before tumor formation and at the end of the experiment. (**G**) Serum succinate concentrations in healthy subjects and HCC patients. (**H**) Receiver operating characteristic curve of the serum succinate level for discriminating HCC patients. Data are presented as mean ± SD. *p < 0.05, **p < 0.01

To investigate the pro-tumorigenic mechanism of succinate *in vivo*, we established a subcutaneous HCC tumor model and conducted functional validation through exogenous succinate administration and pharmacological intervention with a GPR91-specific inhibitor (compound 4c). Compared to the control group, the succinate-treated group exhibited significantly higher HCC tumor growth, as evidenced by increased tumor volume and weight (Fig. 8A–C). However, compound 4c effectively reversed the *in vivo* tumor-promoting effects of succinate. IHC of tumor sections (Fig. 8D) revealed that CD163^+^ cells were significantly increased in the succinate treatment group. This finding suggests that succinate treatment promoted the polarization of TAMs toward the M2 phenotype through GPR91 receptor activation. Additionally, succinate modulated TAM activation status and enhanced their pro-tumorigenic functions. However, these pro-tumorigenic effects of succinate were significantly suppressed by the GPR91 inhibitor. The infiltration level of M2 macrophages in the subcutaneous HCC tumor tissues correlated positively with tumor weight (r = 0.81, *p* < 0.01) (Fig. 8E). This finding further confirmed the tumor-promoting role of M2-polarized TAMs. These results demonstrate that succinate promotes HCC progression by inducing M2 macrophage polarization in a GPR91-dependent manner.

### 3.11 Serum Succinate is a Potential Diagnostic Biomarker for HCC

Following the detection of elevated succinate levels in HCC tissues, we investigated the association between serum succinate levels and HCC risk. Initially, we examined the dynamic changes in serum succinate levels in the subcutaneous HCC tumor model mice. Serum succinate levels were significantly higher in tumor-bearing mice at the end of the experiment compared to pre-tumor levels (Fig. 8F). Subsequently, we analyzed serum samples from 54 HCC patients and 18 healthy subjects to validate these findings. As shown in Fig. 8G, the serum succinate levels in HCC patients were significantly higher than those in the healthy control group. To assess the diagnostic value of the serum succinate level, we conducted receiver operating characteristic curve analysis. Serum succinate demonstrated good diagnostic efficacy in identifying HCC patients with an area under the curve (AUC) value of 0.815 (Fig. 8H). These results indicate that elevated serum succinate levels correlate closely with HCC progression and may serve as a potential biomarker for HCC.

## 4 Discussion

The dynamic interaction between immune cells and tumor cells within the TME is intricate and regulates the biological behavior of tumors and their response to therapy [23]. Tumor cells reprogram the TME and regulate immune cell functions and phenotypes through metabolic pathway alterations, facilitating immune evasion and tumor progression [24]. The present study reveals a novel metabolic-immunological regulatory axis in HCC progression involving SDHA deficiency-mediated accumulation of succinate, which subsequently promotes macrophage polarization into the M2 phenotype.

This investigation systematically assessed immune cell infiltration in the HCC-TME, revealing elevated infiltration of M2 macrophages and resting CD4^+^ memory T cells. Notably, increased M2 macrophage infiltration correlated significantly with poor HCC patients survival. Although higher infiltration of resting CD4^+^ memory T cells suggested improved survival outcomes, the results lacked statistical significance. Additionally, deceased HCC patients exhibited higher M2 macrophage levels compared to survivors, consistent with previous research [25]. M2 macrophages within the TME facilitate tumor progression and metastasis by inhibiting T cell activity and promoting tumor immune evasion through the secretion of anti-inflammatory cytokines and chemokines, including interleukin-10 (IL-10) and TGF-β [26]. To investigate the underlying mechanisms, we identified 104 metabolism-related DEGs enriched in mitochondrial functions. Mitochondria, serving as cellular powerhouses and metabolic centers, are fundamentally linked with tumor metabolic reprogramming [27], substantially influencing tumor development and progression and macrophage infiltration in the TME [28]. Mitochondrial dysfunction directly influences cellular metabolism and contributes to TME remodeling [29]. Impaired mitochondrial function in cancer cells increases aerobic glycolysis, resulting in lactate accumulation and subsequent suppression of anti-tumor immunity [30].

Our findings demonstrate that SDHA is a crucial regulator of tumor metabolism and immune evasion in HCC. SDHA is the core subunit of the SDH complex and catalyzes the oxidation of succinate to fumarate in the TCA cycle [31]. Previous studies have shown that SDHA promotes HCC proliferation [32]. To the best of our knowledge, the present study is the first to report the extrinsic immunomodulatory function of SDHA in succinate-mediated macrophage phenotype switching. SDHA expression was markedly reduced in HCC tissues and inversely correlated with the infiltration status of M2 macrophages. Functional assays confirmed that SDHA silencing induced M2 polarization, whereas its overexpression inhibited M2 polarization. Mechanistically, SDHA deficiency diminishes SDH activity, resulting in elevated levels of succinate [17,32]. Our findings indicate that HCC tissues with higher succinate levels correlate with decreased SDHA expression and enhanced M2 macrophage infiltration. Additionally, serum succinate showed a significant diagnostic value in identifying HCC patients (AUC = 0.815), indicating its potential as a non-invasive diagnostic biomarker for HCC. Thus, monitoring the levels of serum succinate can potentially assist clinicians in disease stratification and treatment response assessment. *In vitro* and *in vivo* experiments further validated the role of succinate in inducing M2 macrophage polarization and their intratumoral infiltration.

Subsequent mechanistic studies revealed that succinate induces M2 polarization of macrophages in a GPR91-dependent manner; however, this process was inhibited by pretreatment with the GPR91-specific inhibitor NF-56-EJ40. These results align with the findings of lung cancer studies where GPR91 knockout blocked similar effects [33]. GPR91, a G protein-coupled receptor, regulates metabolism, immunity, and tumorigenesis through multiple signaling pathways [34]. Several studies have also demonstrated that STAT3 and STAT6 are critical signaling pathways for M2 macrophage polarization [35]. GSEA results indicated that the JAK-STAT pathway is involved in the M2 macrophage polarization process of HCC. Therefore, we performed experiments with a STAT3 inhibitor and found that succinate-induced M2 macrophage polarization requires GPR91/STAT3 signaling. However, other studies have reported that succinate activates TAM through phosphoinositide 3-kinase (PI3K)/hypoxia-inducible factor 1-alpha (HIF-1α) or GPR91/Gq pathways [33,36]. Therefore, we hypothesize that STAT3 may function as a common downstream effector molecule of distinct signaling pathways or may represent synergistic interactions between multiple pathways.

While our study has elucidated the SDHA/succinate/GPR91/STAT3 signaling axis, it is essential to contextualize this finding within the broader pathophysiology of mitochondrial dysfunction in HCC. The SDH complex is a multi-subunit enzyme, and deficiencies in its other components (SDHB, SDHC, and SDHD) are also known to cause succinate accumulation and are implicated in tumorigenesis [37]. Therefore, the identified succinate/GPR91/STAT3 pathway may represent a common mechanism through which SDH-deficient HCC cells recruit M2 macrophages, regardless of the initially affected subunit. Additionally, succinate functions as a pleiotropic metabolite capable of signaling through alternative pathways. Intracellularly, it stabilizes HIF-1α, thereby promoting angiogenesis and the Warburg effect [38]. The potential interaction between this HIF-1α pathway and the paracrine GPR91/STAT3 axis warrants further investigation to fully understand succinate’s role in remodeling the immune microenvironment of HCC.

Because enhanced OXPHOS is a hallmark metabolic feature of M2 macrophage polarization [39], we examined mitochondrial remodeling in succinate-stimulated macrophages. Succinate treatment significantly increased OXPHOS activity; however, these effects were reversed by inhibiting GPR91 or STAT3. This finding indicates that succinate-induced macrophages, similar to typical M2 macrophages, require elevated energy metabolism to maintain polarization. Mechanistically, increased OXPHOS in succinate-induced macrophages may involve several factors. STAT3 activation may directly regulate enhanced mitochondrial function [40]. Moreover, the enhancement of OXPHOS during M2 macrophage polarization may also represent an associated phenomenon. Specifically, the activation of STAT3 may promote the expression of genes related to M2 polarization, and these changes in gene expression may further lead to the upregulation of OXPHOS-related genes, ultimately resulting in enhanced OXPHOS [41]. However, additional detailed experiments are necessary to verify these hypotheses.

This study also revealed that succinate-induced M2 macrophages promote HCC progression by enhancing HCC cell proliferation, migration, and invasion *in vitro* and accelerating HCC tumor growth *in vivo*. Moreover, succinate-treated tumor tissues show increased infiltration of M2 macrophages. In lung cancer, succinate-induced M2 macrophages did not stimulate tumor growth but promoted metastasis through interleukin-6 (IL-6) secretion [33]. This divergence potentially reflects tumor-specific heterogeneity. M2 macrophages produce various cytokines, including IL-6, IL-10, and VEGF [42]. IL-6 contributes to HCC proliferation and sorafenib resistance [43,44]. Therefore, we hypothesize that succinate-induced macrophages secrete HCC-promoting cytokines. However, additional experiments are needed to identify these cytokines and their underlying mechanisms.

This study has several limitations. First, our assessment of immune cell infiltration depends on bioinformatics algorithms such as CIBERSORT; these results represent computational inferences based on transcriptomic data rather than direct experimental measurements. Second, regarding clinical sample analysis, the sample size of serum and tissue specimens remains relatively limited, potentially affecting statistical robustness and restricting detailed analysis across different clinical subgroups. Third, decreased SDH activity may promote M2 macrophage polarization through the synergistic effects of succinate and lactate, as SDH inhibition enhances the Warburg effect, increasing lactate accumulation [45]. However, this potential synergistic mechanism awaits direct validation.

## 5 Conclusions

**Figure 9:**
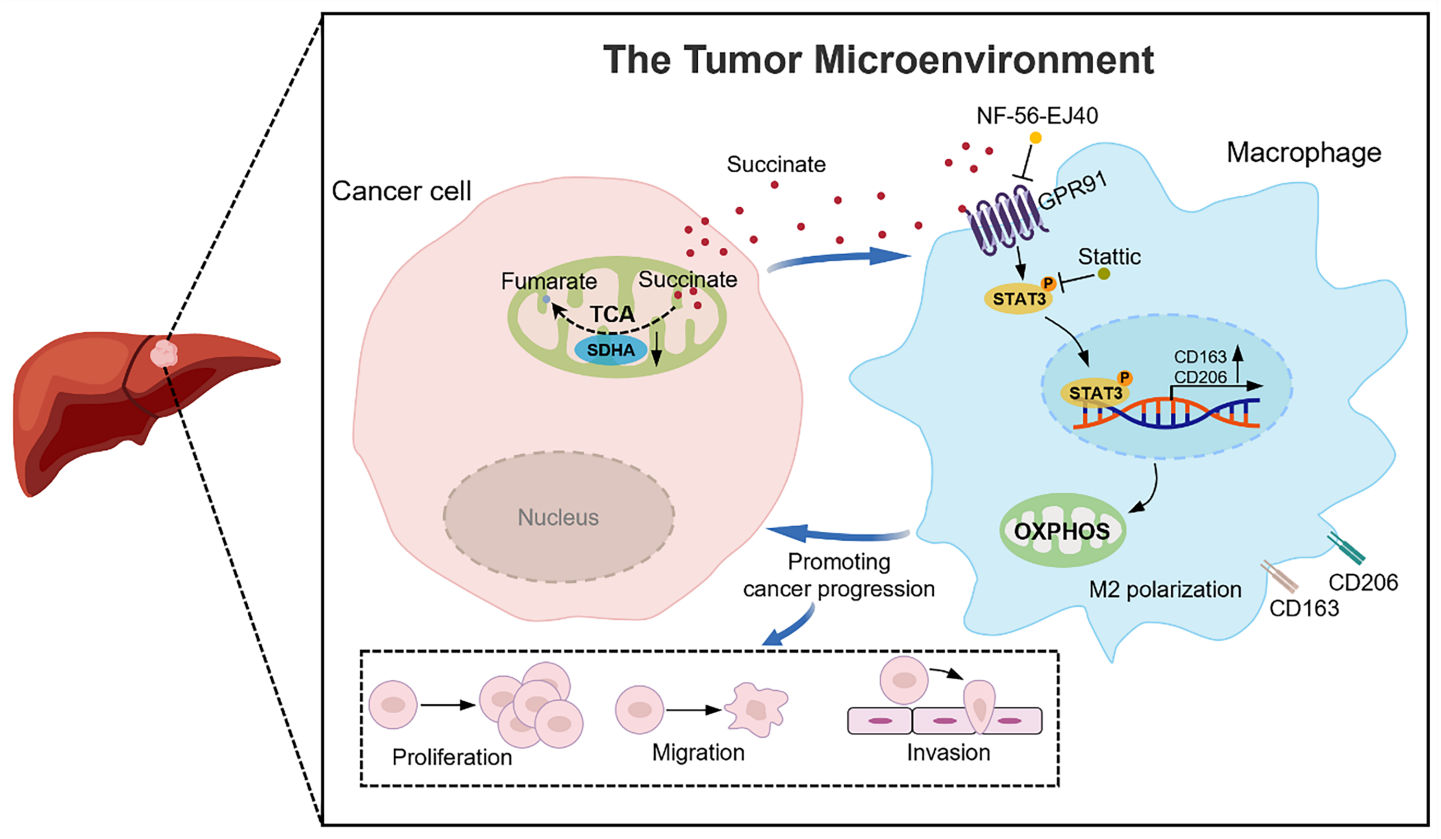
A schematic diagram illustrates that SDHA downregulation in HCC leads to intracellular succinate accumulation and subsequent release into the tumor microenvironment. This tumor-derived succinate induces M2 macrophage polarization through the GPR91/STAT3 pathway to facilitate HCC progression

This study revealed a complex relationship between SDHA deficiency and poor HCC prognosis, along with its underlying mechanisms. Our findings demonstrate that elevated succinate levels resulting from SDHA deficiency promote M2 macrophage polarization and HCC progression through the GPR91/STAT3 signaling pathway (Fig. 9). Additionally, our data indicate that serum succinate is a promising non-invasive diagnostic and/or prognostic biomarker for HCC. These findings establish succinate and GPR91 as potential therapeutic targets for HCC with significant translational implications.

## Supplementary Materials



## Data Availability

The data that support the findings of this study are available from the corresponding author upon reasonable request.

## Supplementary Materials

The supplementary material is available online at https://www.techscience.com/doi/10.32604/or.2025.073179/s1.

## Abbreviations

HCC: Hepatocellular carcinoma
TME: Tumor microenvironment
TAMs: Tumor-associated macrophages
DEGs: Differentially expressed genes
SDH: Succinate dehydrogenase
SDHA: Succinate dehydrogenase complex subunit A
OXPHOS: Oxidative phosphorylation
TCA: Tricarboxylic acid cycle
TCGA: The Cancer Genome Atlas
GEO: The Gene Expression Omnibus
CIBERSORT: Cell-type identification by estimating relative subsets of RNA transcripts
MRGs: Metabolism-related genes
DAVID: The database for annotation, visualization and integrated discovery
STRING: Search tool for the retrieval of interacting genes/proteins
BP: Biological process
CC: Cellular component
MF: Molecular function
GSEA: Gene set enrichment analysis
PPI: Protein-protein interaction
GPR91: G protein-coupled receptor 91
qRT-PCR: Quantitative reverse transcription PCR
IHC: Immunohistochemistry
WB: Western blot
ELISA: Enzyme-linked immunosorbent assay
CM: Conditioned medium
TNF-α: Tumor necrosis factor-alpha
TGF-β: Transforming growth factor-beta
mtDNA: Mitochondrial DNA
MMP: Mitochondrial membrane potential
ROS: Reactive oxygen species
PBS: Phosphate-buffered saline
SOD: Superoxide dismutase
ATP: Adenosine triphosphate
SD: Standard deviation
SEM: Standard error of the mean
OS: Overall survival
ACADL: Long-chain acyl-CoA dehydrogenase
SORD: Sorbitol dehydrogenase
SUCNR1: Succinate receptor 1
CD163: Cluster of differentiation 163
VEGF: Vascular endothelial growth factor
CD206: Cluster of differentiation 206
CD86: Cluster of differentiation 86
IHC: Immunohistochemistry
JAK: Janus kinase
STAT3: Signal transducer and activator of transcription 3
TOMM20: Translocase of outer mitochondrial membrane 20
AUC: Area under the curve
PI3K: Phosphoinositide 3-kinase
HIF-1α: Hypoxia-inducible factor 1-alpha
IL-10: Interleukin-10
IL-6: Interleukin-6
