# Supplementary material for: SDHA Deficiency in Hepatocellular Carcinoma Promotes Tumor Progression through Succinate-Induced M2 Macrophage Polarization
Source: Oncol Res. 2026 Jan 19;34(2):25. doi: 10.32604/or.2025.073179 (PMC12848743; doi:10.32604/or.2025.073179)
Supplement: Supplementary file 1 [file OncolRes-34-73179-s001.docx]

**Table S1:** Primers in this study

| Gene | Forward Primer (5’-3’) | Reverse Primer (5’-3’) |
| --- | --- | --- |
| SDHA | ACGGGTCCATCCATCGCATA | AGAATGCCTCCCTCTCCACGA |
| SORD | CCAAAGCAATGGGAGCAGC | CCAGAGCGAGTGGCGTAGAT |
| ACADL | CAAGGTGTTCATCAGTAATGGGTC | TAGTTCTGCGGTATCCTGGGCT |
| CD206 | TCCTTGTGGGATTGTCCTGC | AAGCCGCTGTCTCTGTCTTC |
| CD163 | AGGGACGTGGCTGTGGATAA | TCCAAAACCCAGAAGACGCAT |
| CD86 | TGTTTCCGTGGAGACGCAAG | TTGAGCCTTTGTAAATGGGCA |
| VEGF | AGGGCAGAATCATCACGAAGT | AGGGTCTCGATTGGATGGCA3 |
| TNF-α | ACCCTCACACTCACAAACCA | ATAGCAAATCGGCTGACGGT |
| β-actin | GGCTGTATTCCCCTCCATCG | CCAGTTGGTAACAATGCCATGT |

**Table S2:** Primers in this study

| Gene | Forward Primer (5’-3’) | Reverse Primer (5’-3’) |
| --- | --- | --- |
| H-MT-CO2 (2) | CCGTCTGAACTATCCTGCCC | AAGATTAGTCCGCCGTAGTCG |
| H-ACTIN | CACCCAGCACAATGAAGATCAAGAT | CCAGTTTTTAAATCCTGAGTCAAGC |


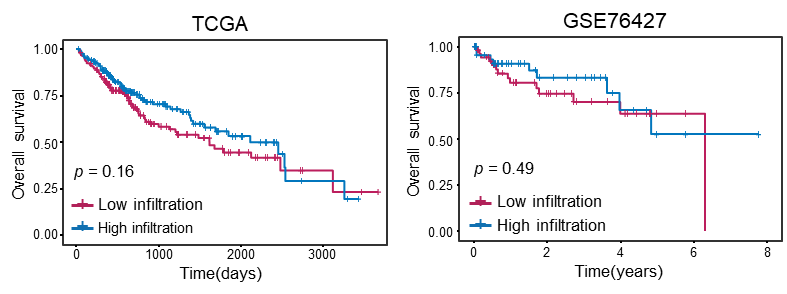


**Figure S1:** Impact of resting CD4^+^ memory T cell infiltration levels on the overall survival of HCC patients.


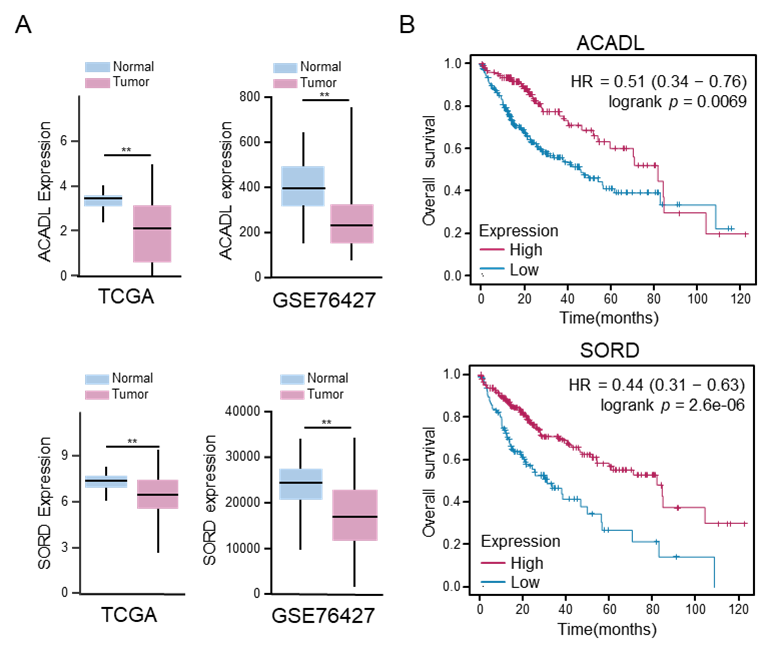


Figure S2: Expression levels and prognostic effects of ACADL and SORD in HCC. (A) Comparison of ACADL and SORD expression between HCC and normal tissues in the TCGA cohort from the TIMER 2.0 database and the GSE76427 cohort. (B) Impact of ACADL and SORD mRNA levels on the overall survival of HCC patients from the Kaplan-Meier plotter database. Data are presented as mean ± SD. ***p* < 0.01.


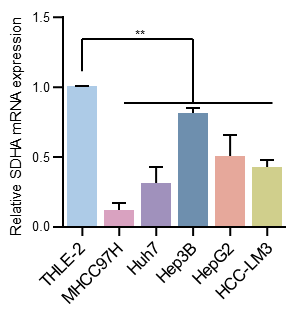


**Figure S3:** Relative mRNA expression of SDHA in liver cells and various HCC cell lines was detected by qRT-PCR. Data are presented as mean ± SD. ***p* < 0.01.


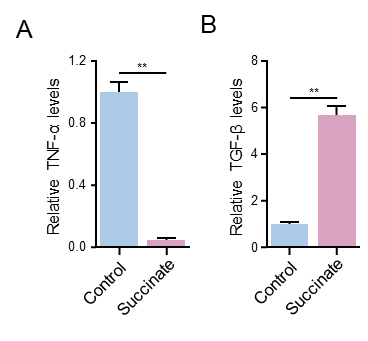


**Figure S4:** Succinate induces macrophage M2 polarization. THP-1(Mφ) cells were treated with or without succinate (1.5 mM) for 48 h. (**A, B**) Relative concentration of TNF-α and TGF-β was assessed by ELISA. Data are presented as mean ± SD. ***p* < 0.01.


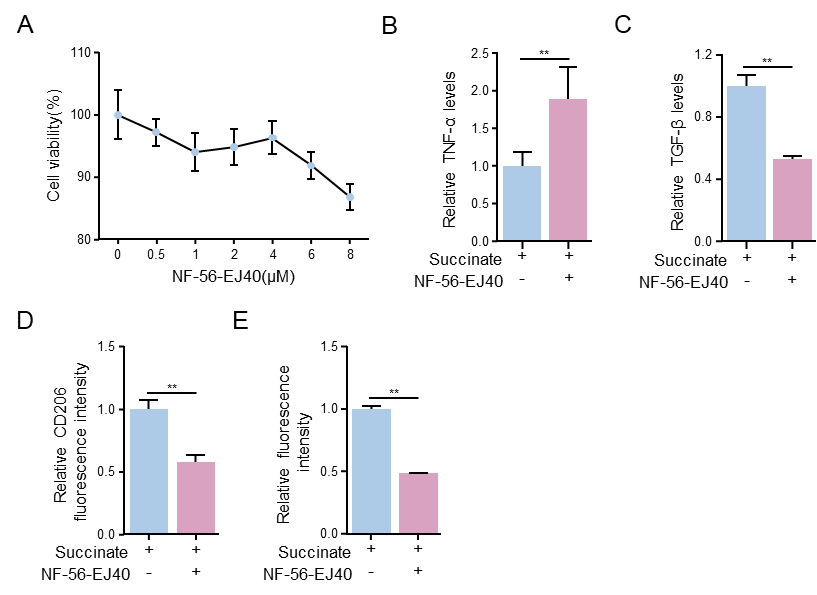


Figure S5: Succinate-induced M2 macrophage polarization depends on the membrane receptor GPR91. HP-1(Mφ) cells were pretreated with or without the GPR91 inhibitor NF-56-EJ40 (4 μM) for 6 h, followed by exposure to succinate (1.5 mM) for 48 h. (A) Detection of cell viability in THP-1(Mφ) cells treated with varying concentrations of NF-56-EJ40 for 48 h. (B, C) Relative concentration of TNF-α and TGF-β was assessed by ELISA. (D) Quantitative analysis of representative immunofluorescence images. (E) Quantitative analysis of changes in CD206^+^ macrophages by flow cytometry. Data are presented as mean ± SD. THP-1(Mφ), macrophages induced from THP-1 cells. ***p* < 0.01.


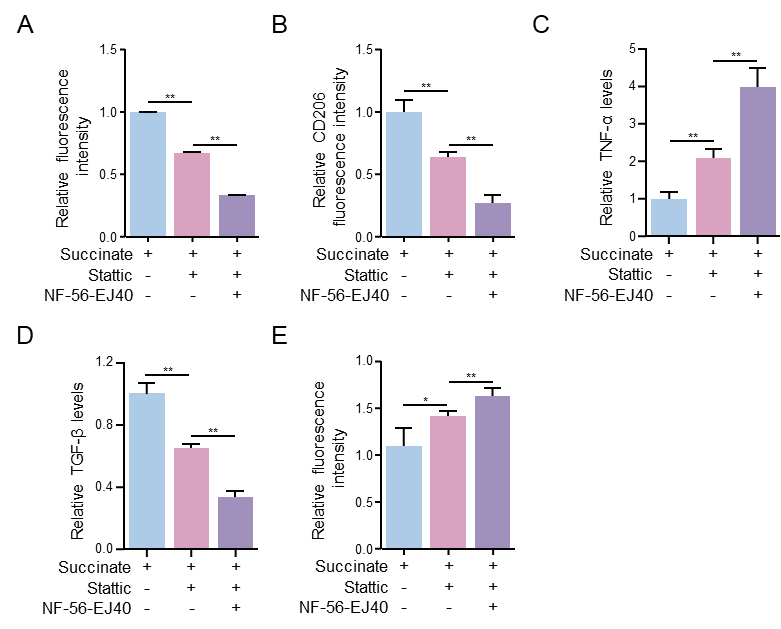


Figure S6: Succinate induces M2 macrophage polarization and enhances mitochondrial function *via* GPR91/STAT3 activation. THP-1(Mφ) cells were pretreated with the STAT3 inhibitor Stattic (5 μM) alone or in combination with the GPR91 inhibitor NF-56-EJ40 (4 μM) for 6 h, followed by exposure to succinate (1.5 mM) for 48 h. (A) Quantitative analysis of changes in CD206^+^ macrophages by flow cytometry. (B) Quantitative analysis of representative immunofluorescence images. (C, D) Relative concentration of TNF-α and TGF-β was assessed by ELISA. (E) Quantitative analysis of ROS by flow cytometry. Data are presented as mean ± SD. **p* < 0.05, ***p* < 0.01.
